# Nanoscale organization of the endogenous ASC speck

**DOI:** 10.1016/j.isci.2023.108382

**Published:** 2023-11-02

**Authors:** Ivo M. Glück, Grusha Primal Mathias, Sebastian Strauss, Virgile Rat, Irene Gialdini, Thomas Sebastian Ebert, Che Stafford, Ganesh Agam, Suliana Manley, Veit Hornung, Ralf Jungmann, Christian Sieben, Don C. Lamb

**Affiliations:** 1 Department of Chemistry, Ludwig Maximilians-Universität München, Butenandtstraße 5-13, 81377 München, Germany; 2 Center for Nano Science (CENS), Ludwig Maximilians-Universität München, Butenandtstraße 5-13, 81377 München, Germany; 3 Faculty of Physics and Center for Nanoscience, Ludwig Maximilian University, Munich, Germany; 4 Max Planck Institute of Biochemistry, Martinsried, Germany; 5 Gene Center and Department of Biochemistry, Ludwig-Maximilians-Universität, Munich, Germany; 6 Laboratory of Experimental Biophysics, École Polytechnique Fédérale de Lausanne, BSP 427 (Cubotron UNIL), Rte de la Sorge, CH-1015 Lausanne, Switzerland

**Keywords:** Immunology, Biotechnology, Cell biology

## Abstract

The NLRP3 inflammasome is a central component of the innate immune system. Its activation leads to formation of the ASC speck, a supramolecular assembly of the inflammasome adaptor protein ASC. Different models, based on ASC overexpression, have been proposed for the structure of the ASC speck. Using dual-color 3D super-resolution imaging (dSTORM and DNA-PAINT), we visualized the ASC speck structure following NLRP3 inflammasome activation using endogenous ASC expression. A complete structure was only obtainable by labeling with both anti-ASC antibodies and nanobodies. The complex varies in diameter between ∼800 and 1000 nm, and is composed of a dense core with emerging filaments. Dual-color confocal fluorescence microscopy indicated that the ASC speck does not colocalize with the microtubule-organizing center at late time points after Nigericin stimulation. From super-resolution images of whole cells, the ASC specks were sorted into a pseudo-time sequence indicating that they become denser but not larger during formation.

## Highlights

• Structure of ASC speck investigated under conditions of endogenous ASC expression
• Combined antibody and nanobody labeling required to obtain complete ASC structure
• The ASC speck is roughly spherical with dense core and emerging filaments
• The ASC speck becomes denser but not larger during its formation

## Graphical abstract

**Figure:**
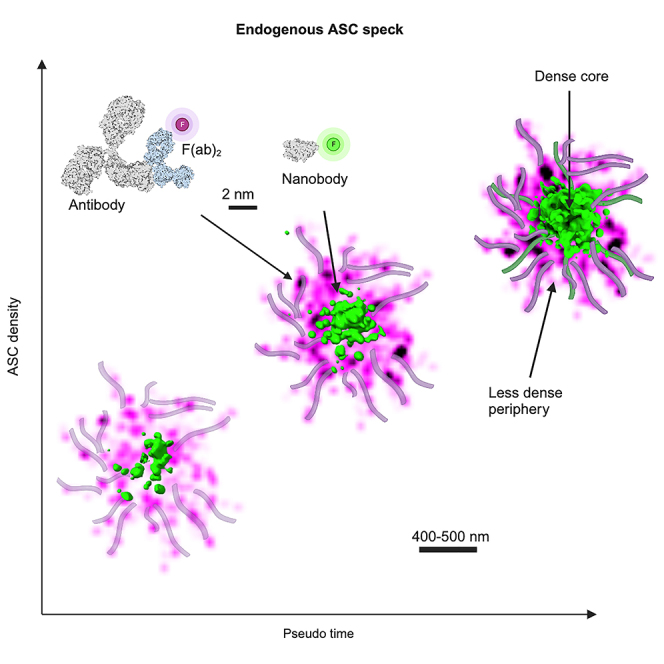

## Introduction

Inflammasomes are a class of large, multiprotein complexes that assemble upon activation of cellular pattern recognition receptors (PRRs).^1^ Being part of the innate immune system, inflammasomes can sense the presence of non-self biomolecules or the perturbation of cellular homeostasis. The key components include a sensor protein, the adaptor protein apoptosis-associated speck-like protein containing a caspase activation and recruitment domain (ASC)^2^^,^^3^ and the inflammatory caspase 1 (CASP1).^4^^,^^5^^,^^6^ The largest group of sensor proteins belongs to the family of nucleotide-binding oligomerization domain-like receptors (NLRs). Among these, NLRP3 (NOD-, LRR-, and pyrin domain-containing protein 3) has been shown to play a critical role in many infections as well as under sterile inflammatory conditions. Although the molecular mode of action remains to be elucidated, potassium efflux, as it occurs in the context of membrane damage, appears to be a key signal upstream of NLRP3 activation.^7^ NLRP3 activation triggers recruitment of ASC and CASP1 leading to a single, micrometer-sized assembly. For the structure of ASC within the inflammasome, Masumoto et al. coined the term “ASC speck”.^3^^,^^8^ ASC is composed of two interaction domains connected by a semi-flexible linker: a pyrin domain (PYD)^9^ and a caspase activation and recruitment domain (CARD).^10^^,^^11^ The individual domains have a high tendency for homotypic interactions due to their complementarity in structure and charge, leading to the assembly of the large multiprotein inflammasome complex.^12^^,^^13^^,^^14^^,^^15^

The details of how the ASC speck is organized are the subject of intensive research. ASC (22 kDa) is soluble at low pH and in a chaotropic solution, but at physiological pH, the protein can assemble into filaments as observed *in vitro* by solid-state NMR spectroscopy and electron microscopy (EM).^16^^,^^17^^,^^18^^,^^19^^,^^20^^,^^21^ The ASC speck can also be purified from inflammasome-activated cells expressing ASC endogenously, where it was found to take on diverse morphologies, including a star-shaped assembly,^8^ isolated filaments, or amorphous aggregates potentially composed of intertwined filaments.^19^^,^^22^ In cells, ASC specks were visualized by microscopy using immunofluorescence or expression of ASC tagged with a fluorescent protein. Upon overexpression, the resulting speck was typically much larger than 1 μm and occasionally showed filaments protruding from the edge of the structure.^20^^,^^22^^,^^23^^,^^24^^,^^25^ In contrast, diffraction-limited immunofluorescence imaging of the endogenous ASC speck revealed a spot of about 1 μm in diameter. Due to the propensity of ASC to self-assemble, it is unclear whether the endogenous structure resembles the complexes observed *in vitro* or upon ASC overexpression. Interestingly, one approach to labeling ASC using an EGFP-labeled nanobody directed against the CARD domain revealed an intermediate, filamentous structure during speck formation, but no ASC specks were observed.^26^ Higher resolution *in situ* studies, either by EM^27^^,^^28^ or super-resolution fluorescence STED (stimulated emission depletion) microscopy,^29^ resolved the endogenous speck as an amorphous aggregate potentially made up of intertwined filaments. In contrast, other studies describe the ASC speck as a hollow, ring-shaped complex^3^^,^^6^^,^^23^^,^^30^^,^^31^^,^^32^^,^^33^^,^^34^^,^^35^^,^^36^^,^^37^^,^^38^ and, based on this observation, different models for ASC speck and inflammasome formation have been proposed.^34^^,^^35^^,^^39^^,^^40^^,^^41^^,^^42^ Despite its relevance for understanding inflammasome formation, the nanoscale organization of the endogenous ASC speck remains controversial.

Here, we performed a systematic study of fluorescence labeling strategies, and used quantitative widefield microscopy, confocal microscopy and single-molecule localization microscopy (SMLM) (i.e., dual-color 3D direct stochastic optical reconstruction microscopy (dSTORM) and DNA-PAINT) to investigate the organization of the endogenous ASC speck. We chose Nigericin treatment to trigger ASC speck formation in our studies as it is the standard protocol used for NLRP3 activation in the field.^43^ Our data resolved filaments protruding from a dense core of the endogenous ASC speck, supporting the amorphous nature of the complex. To address the hypothesis suggested in the literature that the microtubule-organizing center (MTOC) is the cellular location of NLRP3 inflammasome formation after Nigericin treatment,^44^ we performed immunofluorescence staining of the centrosomal marker pericentrin and the ASC speck. Our data suggests that, after prolonged periods of NLRP3 stimulation, the speck does not colocalize with the MTOC. By using two complementary labeling approaches comparing antibody-with nanobody-labeled ASC, we could probe the organization of and density differences within the ASC speck. We found that nanobodies labeled the center of the speck while the antibody was predominantly detected in the periphery of the complex, occasionally exhibiting a hollow center. Thus, our results reconcile the disparate structures reported in the literature. Furthermore, we analyzed the redistribution of ASC into the speck using single-cell analysis, allowing us to sort specks with respect to the degree of ASC recruitment. Our results indicate that endogenous specks mainly become denser but only slightly larger during inflammasome assembly.

## Results

### Endogenous specks vary strongly in ASC content

**Figure 1:**
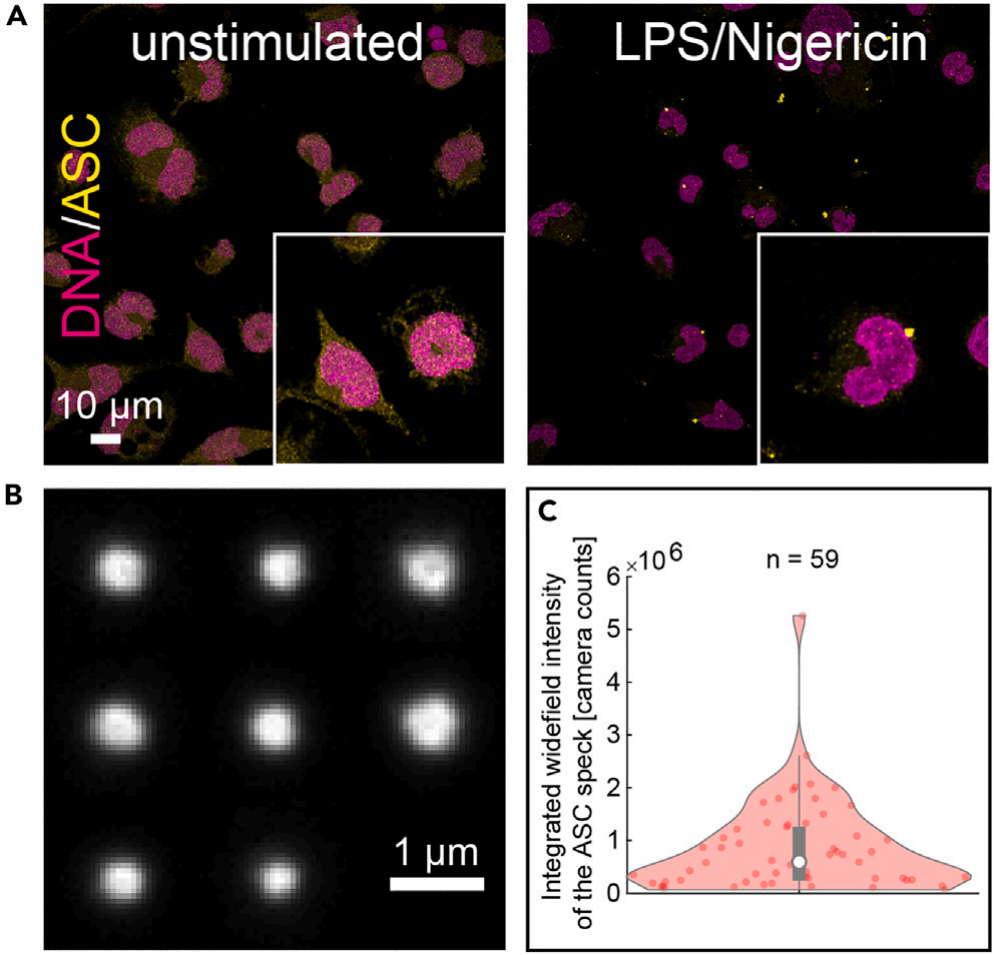
LPS + Nigericin stimulation of THP-1 caspase-1 knock-out cells induces ASC speck formation with variable size and ASC content (A) Confocal images (maximum projections) of fluorescently labeled ASC (shown in yellow) in unstimulated (left panel) and in LPS + Nigericin-stimulated THP-1 caspase-1 knock-out cells in the presence of Z-VAD-FMK (right panel). The formation of ASC specks after stimulation is clearly visible. DNA was stained using DAPI (shown in magenta). (B) A montage of ASC specks immunostained using a primary antibody and a secondary Alexa Fluor 647-conjugated F(ab’)_2_ fragment and imaged by diffraction-limited widefield illumination with 60× magnification. The speck size varies and the appearance of a darker center is observed for some of the specks. (C) A violin plot of the integrated widefield intensity of ASC specks imaged as described for (B). There is a large variation in the intensity, which serves as an indicator for the amount of protein incorporated into the speck. Pooled data from three independent cell preparations is shown. See also Figure S1.

**Figure 2:**
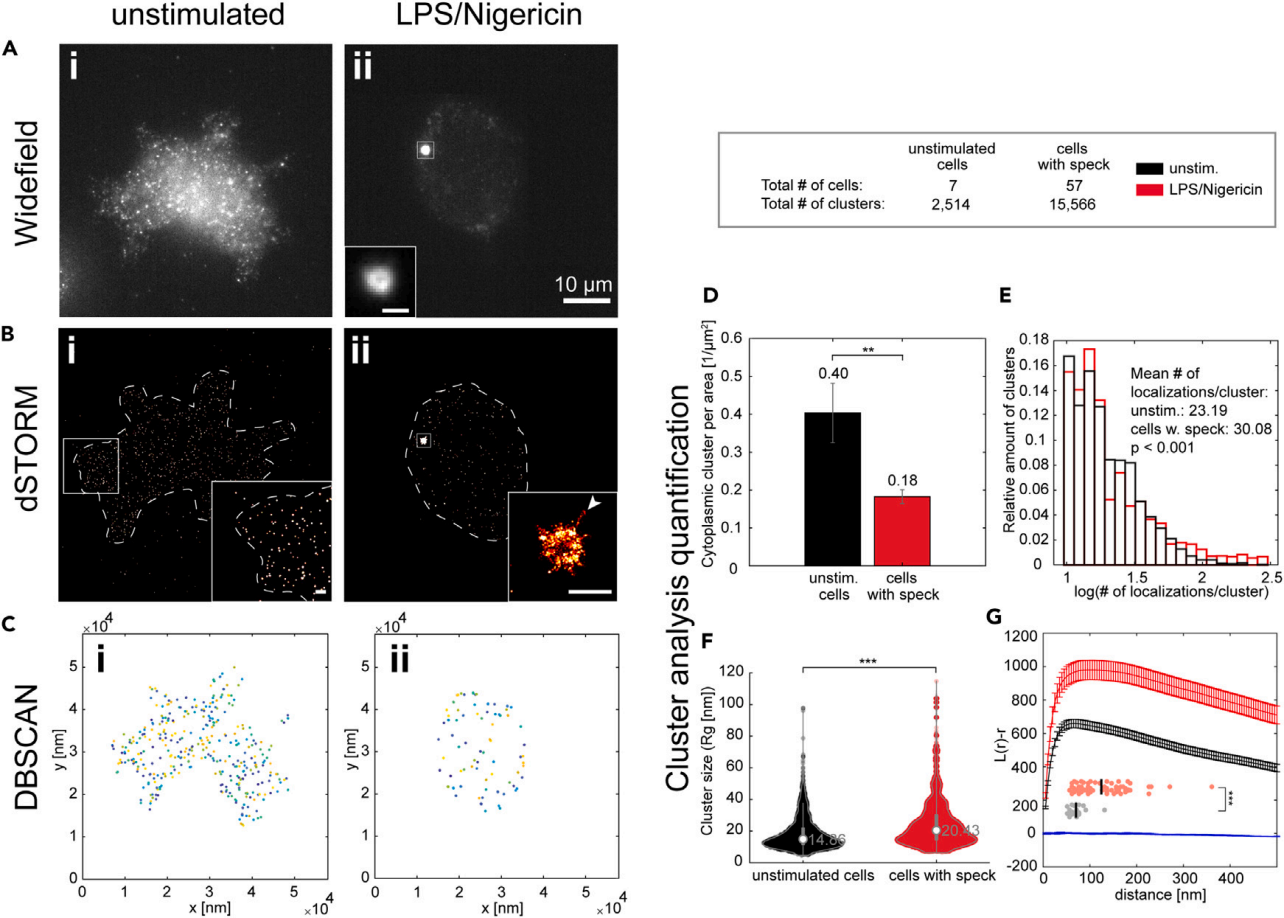
Whole-cell dSTORM super-resolution microscopy of endogenous ASC in THP-1 caspase-1 knock-out cells (A and B) The distribution of ASC in non-stimulated, caspase-1 knock-out cells (i) and the redistribution of ASC into the speck after stimulation with LPS and Nigericin in the presence of Z-VAD-FMK (ii) observed using diffraction-limited widefield imaging (A) and dSTORM (B). dSTORM additionally resolves filaments protruding from the speck core (one example is highlighted by the arrow). Scale bars in insets correspond to 1 μm. (C) DBSCAN clustering analysis of cytosolic ASC localizations (non-speck bound ASC) in the unstimulated (i) and LPS and Nigericin activated (ii) cells. The analysis confirms depletion of the protein from the cytosol after speck formation. Color coding is used to distinguish individual clusters. (D–F) The results of the DBSCAN analysis are shown. (D) The non-speck incorporated ASC cluster density decreases in cells exhibiting a speck due to recruitment of the protein into the speck. The error bars indicate the standard error of the mean calculated from the analysis results of individual cells. (E and F) The distribution of the number of localizations (E) and violin plots of the radius of gyration (*R*_*g*_) (F) for unstimulated cells and cells forming a speck. The distributions are similar with an additional population observable in speck-producing cells with more localizations and a larger size compared to non-stimulated cells. White circles in the violin plots indicate the median of the distribution. The statistical significance in D–F was assessed by a Two-Sample Kolmogorov-Smirnov Test (∗) p < 0.05, (∗∗) p < 0.01, (∗∗∗) p < 0.001. (G) A Ripley’s K analysis of the ASC clusters. The Ripley’s K function confirms the decrease in cluster density after speck formation as well as the increase in cluster size. The curves show the mean ± SD of 100 regions of interest (ROI) from 40 cells imaged from three independent experiments. For the estimation of the curve’s maximum (G, lower part), we measured the maximum of the Ripley's K function of the individual ROIs and calculated the mean of the values. For comparison, the same data were taken and the positions of the individual localizations were randomized at the same density and analyzed accordingly. The statistical significance was assessed using a one-sided t-test (∗∗) p < 0.005, (∗∗∗) p < 0.001. Data on unstimulated cells was obtained from a single experiment. Data on cells with speck was obtained from three independent cell preparations. See also Figures S2 and S3.

We investigated the organization of the endogenous ASC speck in THP-1 cells. To maximize the number of cells showing an ASC speck in our study, we used caspase-1 knockout cells additionally treated with a pan-caspase inhibitor (Z-VAD-FMK) as caspase activation upon inflammasome activation leads to cell death. The cells were primed using lipopolysaccharide (LPS)^45^ followed by stimulation with the bacterial, potassium-efflux-inducing ionophore Nigericin.^43^ After 90 min, the cells were fixed using paraformaldehyde and stained with a primary monoclonal antibody against ASC in combination with a secondary Alexa Fluor 647-tagged F(ab’)_2_ fragment. To gain an initial insight into the distribution of ASC within the cell, we imaged the cells at low magnification (10x) using confocal microscopy. In unstimulated cells, ASC was distributed throughout the cell, including the nucleus (Figure 1A, left panel, Figure S1A). Upon NLRP3 inflammasome activation, ASC relocated into the characteristic single perinuclear speck in about 30% of the cells (Figure 1A, right panel).^46^ We could also observe extracellular specks as previously reported^22^^,^^27^ (Figure S1B). Next, we used high magnification (60x) widefield microscopy to image 59 individual ASC specks, which appear as spherical complexes ranging in size between approximately 0.5–1 μm diameter (Figure 1B). Analysis of the integrated intensity of the ASC signal revealed that the amount of incorporated ASC, as reflected by antibody binding, can vary by almost one order of magnitude (Figure 1C). The widefield microscopy images also confirmed that ASC is distributed throughout unstimulated cells (Figure 2Ai) and that, at the sensitivity level of the here applied diffraction-limited widefield fluorescence microscopy, the cytoplasmic ASC in cells showing a speck appeared almost completely redistributed into a single, bright ASC spot (Figure 2Aii). These observations are consistent with previous reports of the ASC speck upon activation of the NLRP3 inflammasome.^47^

### Non-speck incorporated ASC clusters maintain their size distribution during ASC speck formation

To study the distribution of ASC at the nanoscale, we used dSTORM.^48^ Reconstructed dSTORM images further confirmed the cytoplasmic ASC distribution in unstimulated cells and its redistribution into a single speck after inflammasome activation. Interestingly, the increased detection sensitivity of dSTORM allowed us to visualize a previously undetected, non-speck-bound ASC population in the cytoplasm of activated cells (Figure 2B). We performed a clustering analysis based on the local density of molecules, which distinguishes cytoplasmic ASC clusters from background localizations (Figures S2 and S3). We used a density-based spatial clustering of applications with noise (DBSCAN) analysis^49^ (Figure 2C), which revealed a decrease in the cluster density from an average of 0.40 clusters per μm^2^ in unstimulated cells to 0.18 clusters per μm^2^ in cells showing a speck (Figure 2D). This is consistent with the observed ASC recruitment (Figure 2Aii). Next, from the detected clusters, we investigated the number of localizations per cluster and the cluster size by calculating the radius of gyration (*R*_*g*_). We found the size of non-speck incorporated clusters to be very similar between unstimulated and stimulated cells (*R*_*g*_ < 20 nm and localizations/cluster <60) (Figures 2E and 2F). However, in speck-containing cells, an additional cytoplasmic cluster population appears with a larger size and more localizations (*R*_*g*_ 20–80 nm and localizations/cluster >60). We also applied a Ripley’s K clustering analysis^50^ as an alternative approach for characterizing the spatial distribution of non-speck incorporated ASC. This analysis (Figure 2G) confirmed the decrease in cytoplasmic cluster density in cells containing a speck as shown by the shift of the amplitude of the obtained L(r)-r curve toward higher values and, in addition, an increase of the L(r)-r maximum value consistent with a small population of larger clusters.

### Super-resolution microscopy reveals filaments extruding from endogenous ASC specks

**Figure 3:**
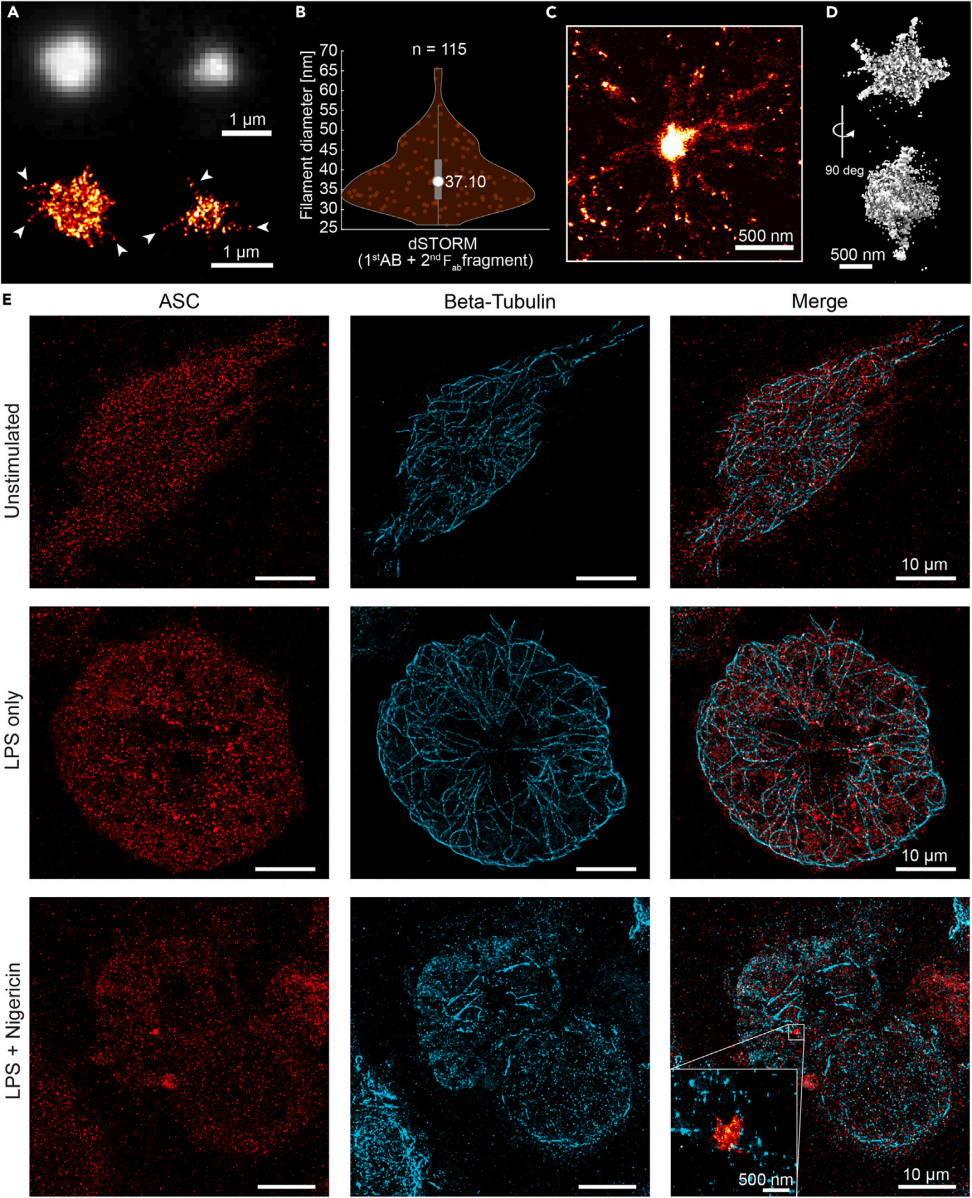
Super-resolution imaging of the endogenous ASC speck and microtubuli (A) Two ASC specks of different sizes stained with primary antibody and secondary F(ab’)_2_ fragment in THP-1 caspase-1 knock-out cells in the presence of Z-VAD-FMK are shown after diffraction-limited widefield imaging (upper images) together with their 2D dSTORM reconstructions (lower images). Scale bars: 1 μm. Note the filaments detectable by dSTORM imaging, protruding from the speck core (several examples are highlighted by the arrows). (B) The distribution of ASC filament diameters as measured in 2D dSTORM reconstructions on multiple cross-sections per filament. The circle in the violin plot indicates the median of the distribution. Data were obtained on three independent cell preparations and 18 individual specks. (C) An ASC speck in BlaER1 cells imaged using DNA-PAINT super-resolution microscopy. Filaments are clearly observed reaching out from a dense core. Scale bar: 500 nm. Note that the filaments appear thicker in this experiment due to the decreased localization precision of out-of-focus localizations. (D) A 3D dSTORM reconstruction of a single ASC speck stained with primary antibody and secondary F(ab’)_2_ fragment in THP-1 caspase-1 knock-out cells in the presence of Z-VAD-FMK. Scale bar: 500 nm. (E) Dual-color DNA-PAINT images of ASC and β-tubulin. ASC distribution throughout the cell is observed for unstimulated and LPS only stimulated cells. After stimulation with LPS and Nigericin in the presence of Z-VAD-FMK, ASC redistribution into the speck is observed. Whereas an intact microtubule network is observed in unstimulated and LPS only stimulated cells, LPS and Nigericin treatment leads to an almost complete disassembly of the microtubule network. For the depicted example of an ASC speck, an association of ASC filaments with the microtubule network is imaginable (insert, lower right panel). Scale bars: 10 μm. See also Figures S4–S7 and Videos S1 and S2.

Next, we investigated the nanoscale organization of the ASC speck itself using SMLM.^51^^,^^52^ We labeled endogenous ASC using a primary antibody in combination with a secondary F(ab’)_2_ fragment. The antibody is known to bind to the CARD domain of ASC. A large proportion of the specks appeared as round, amorphous structures with a diameter of about 1 μm exhibiting a rough surface with short protrusions (Figure S4A). Interestingly, for a large number of the specks, the higher resolution obtained by dSTORM imaging resolved ASC filaments reaching out from the dense core of the speck (Figure 3A). The number of clearly resolved filaments per structure varied but we rarely observed more than ∼10. We measured the diameter of the filaments using a Gaussian fitting of the localization density profile of multiple cross sections along each filament. The filament diameter was derived from the full width at half maximum of the localization density profile, where we found a median value of 37.1 nm (Figure 3B). Considering the size of a primary/secondary F(ab’)_2_ antibody complex (∼11 nm)^53^ used for labeling and the experimental localization precision of ∼10 nm (see STAR Methods), the measured value corresponds to an actual thickness of the filament of ∼15 nm. When compared to values obtained for filaments formed by ASC *in vitro* and studied by EM (16 nm),^17^ this value indicates that the majority of filaments are isolated single filaments.

To validate the presence of filaments in ASC specks, we also imaged the endogenous ASC specks using DNA-PAINT super-resolution microscopy.^54^^,^^55^ Here, we used BlaER1 cells, transdifferentiated into monocytes/macrophages in which ASC was endogenously tagged with TagRFP.^56^^,^^57^^,^^58^ We note that these cells still express caspase-1, although a caspase inhibitor was added to the cell medium. This alternative system again revealed the morphology of the endogenous ASC speck including filaments and a dense core, suggesting that these features do not depend on the expression of caspase-1. Strikingly, the filaments were much longer than the ones observed by dSTORM in THP-1 cells (Figures 3C and S4). The emergence of filaments was also observed in dSTORM images recorded in 3D (Figures 3D and S5).

It has been suggested in the literature that ASC is transported to the speck along microtubules.^44^^,^^59^ Hence, the observed filaments could represent ASC on microtubules. To investigate this possibility, we performed two-color 3D DNA-PAINT of ASC and microtubules in THP-1 cells (via labeling of β-tubulin, see STAR Methods). While unstimulated cells and cells only stimulated with LPS have a clear microtubule network (Figure 3E upper two panels), the microtubule network is completely disassembled in the majority of cells displaying an ASC speck when stimulated with both LPS and Nigericin (Figure 3E bottom panel and Figure S6). Occasionally, some residual microtubule network is observable in cells containing an ASC speck. In the case shown in Figure 3E bottom panel, it is possible to imagine that the filaments are in close vicinity to what is still detectable from the microtubules. Although not conclusive, the data would be consistent with ASC filament formation along microtubules but could also be due to transient interactions between ASC and microtubules or cargo proteins. However, the filaments remain part of the ASC speck structure even after microtubules disassemble.

### The ASC speck does not colocalize with the MTOC after 90 minutes of Nigericin stimulation

**Figure 4:**
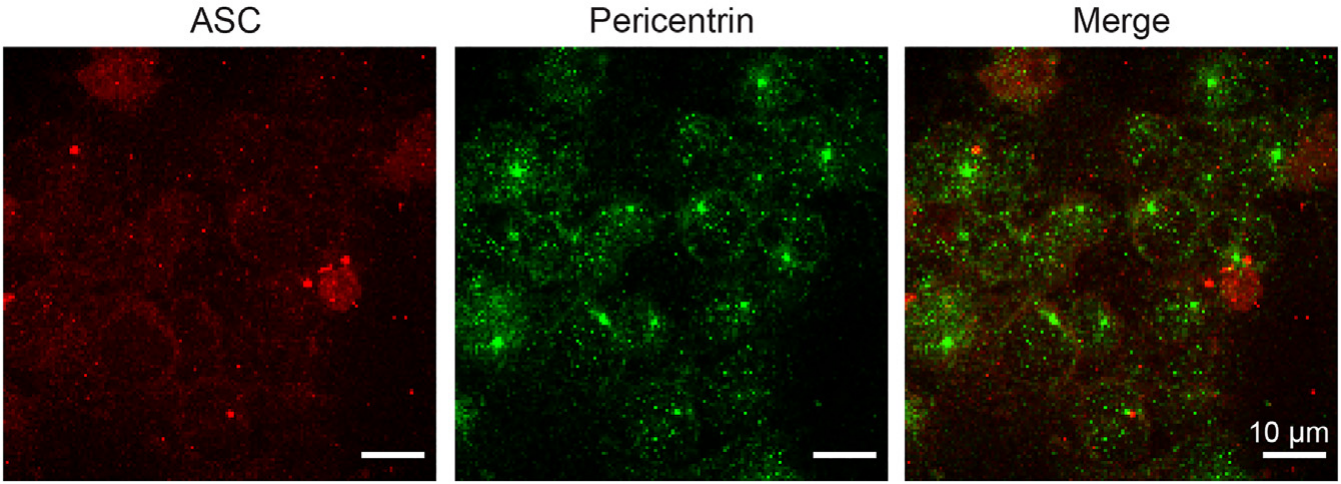
A dual-color confocal spinning-disk image of labeled ASC and pericentrin in THP-1 caspase-1 knock-out cells A representative field of view showing (left) Alexa Fluor Plus 488-antibody-labeled ASC in red, (middle) Alexa 555-labeled-antibodies against pericentrin in green as a marker for the MTOC and (right) a merged image. ASC specks are visible as bright red dots in the ASC channel, which do not colocalize with the MTOCs labeled in green. Cells were stimulated with LPS and Nigericin and the experiments performed in the presence of Z-VAD-FMK. Scale bar: 10 μm.

If ASC is only transported along microtubules, one would expect the ASC speck to form at or near the MTOC as has been suggested previously.^44^^,^^59^^,^^60^ Hence, we performed dual-color spinning-disk confocal microscopy of THP-1 caspase-1 knock-out cells, primed with LPS and stimulated with Nigericin (for 90 min at 10 μM) in the presence of Z-VAD-FMK as before where ASC and pericentrin, an established MTOC marker, were fluorescently labeled. Figure 4 shows a field of view with several cells containing an ASC speck. None of the specks colocalize with the MTOC. In the 48 cells we analyzed that exhibited both an MTOC and ASC speck signal, none of them colocalized when measured using 90-min Nigericin stimulation.

### Super-resolution microscopy reveals distinct morphologies of the endogenous ASC speck

**Figure 5:**
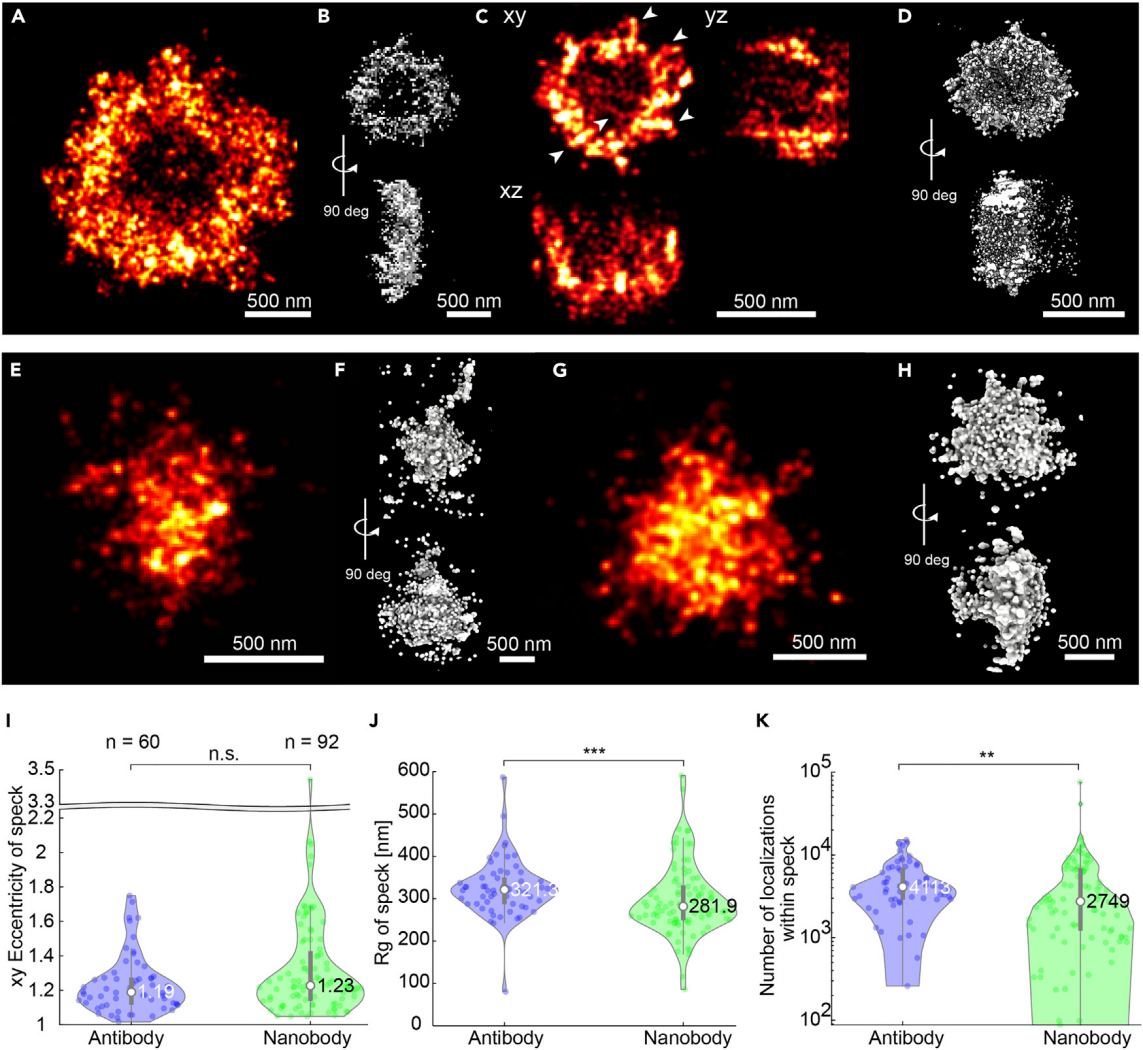
ASC speck morphologies observed using super-resolution imaging ASC specks were formed in THP-1 caspase-1 knock-out cells after LPS and Nigericin stimulation in the presence of Z-VAD-FMK. (A–D) A characteristic 2D reconstruction (A) and a 3D reconstruction of an ASC speck (B) as well as xy, xz, and yz projections of a 3D dSTORM reconstruction (C) and a 3D reconstruction of another ASC speck (D) are shown. Both exhibit a ring-like structure. In (C), the arrows highlight filament-like structures. (E–H) ASC specks stained with anti-ASC nanobodies. 2D (E) and 3D (F) reconstructions of two individual ASC specks imaged by dSTORM. G-H) 2D (G) and 3D (H) representations of an ASC speck imaged using DNA-PAINT. Short filamentous structures are resolvable at the edge of the dense speck core. (I–K) Comparison of the endogenous ASC speck parameters determined using primary antibody and secondary F(ab’)_2_ fragment labeling (blue) and nanobody labeling (green). From the 2D dSTORM images, violin plots of the eccentricity (I), the radius of gyration (J) and the number of localizations (K) were determined. White circles in the violin plots indicate the median of the distribution. The statistical significance was assessed by a two-sided, two-sample Kolmogorov-Smirnov test as indicated: (∗) p < 0.05, (∗∗) p < 0.01, (∗∗∗) p < 0.001. Data on antibody- and nanobody-stained specks were obtained from three and two independent cell preparations, respectively. See also Figures S2, S4, S7, S8, and S9 and Videos S3, S4, S5, and S6.

Next, we investigated the morphology of the endogenous ASC specks in 3D using dSTORM. The specks had an overall spherical shape with filaments occasionally reaching out from a dense core (Figures S5, S7, Videos S1 and S2). For some ASC specks, we observed a ring-like appearance (Figures 5A–5D, S4A, and S7). Some of the structures appeared less extended along the z axis resulting in an overall disk-like shape (Figures 5A and 5B, Videos S3 and S4) while others had a spherical, hollow shape (Figures 5C and 5D, Video S5). We note that, due to the limited imaging depth when using astigmatism for 3D localization, only the central 800 nm region of the specks are shown. This can lead to flattening of larger specks. When focusing on the central region of the speck, in all cases, the signal was not entirely excluded from the center. We hypothesized that local density differences may be responsible for the ring-like signal, perhaps due to the steric exclusion of primary and secondary antibody complexes from the dense center of the structure.

Video S1. Three-dimensional super-resolution rendering of an endogenous ASC speck, related to Figure 2 and Figure S5 3D rendering of an endogenous ASC speck stained with anti-ASC primary antibodies and secondary Alexa Fluor 647-conjugated F(ab’)_2_ fragments and measured using dSTORM. Note the overall spherical appearance characterized by a dense core and protruding filaments. The depicted complex exhibits a diameter of about 1 μm.

Video S2. Three-dimensional super-resolution rendering of an endogenous ASC speck, related to Figure 2 and Figure S5 3D rendering of an endogenous ASC speck stained with anti-ASC primary antibodies and secondary Alexa Fluor 647-conjugated F(ab’)_2_ fragments and measured using dSTORM. Note the overall spherical appearance characterized by a dense core and protruding filaments. The depicted complex exhibits a diameter of about 1 μm.

Video S3. Three-dimensional super-resolution rendering of an endogenous ASC speck, related to Figure 5 3D rendering of an endogenous ASC speck stained with anti-ASC primary antibodies and secondary Alexa Fluor 647-conjugated F(ab’)_2_ fragments and measured using dSTORM. Note the overall ring-like appearance. The depicted complex exhibits a diameter of about 1 μm.

Video S4. Three-dimensional super-resolution rendering of an endogenous ASC speck, related to Figure 5 3D rendering of an endogenous ASC speck stained with anti-ASC primary antibodies and secondary Alexa Fluor 647-conjugated F(ab’)_2_ fragments and measured using dSTORM. Note the overall ring-like appearance. The depicted complex exhibits a diameter of about 1 μm.

Video S5. “Z-stack” super-resolution rendering of an endogenous ASC speck, related to Figure 5 “Z-stack” 3D rendering of an endogenous ASC speck stained with anti-ASC primary antibodies and secondary Alexa Fluor 647-conjugated F(ab’)_2_ fragments and measured using dSTORM. Note the overall ring-like appearance. The depicted complex exhibits a diameter of about 1 μm.

To test this hypothesis, we performed 3D super-resolution imaging using a 3-fold smaller ASC nanobody^26^ to stain endogenous specks. The nanobody interacts with the α1 and α6 helices of the CARD domain of ASC.^26^ Following nanobody labeling and dSTORM imaging, the specks appeared as amorphous structures with no obvious organization (Figures 5E–5H, S8A, and S9). The structure remained spherical (Figure 5I) while the overall size of the specks, determined from the radius of gyration, was smaller (Figure 5J). The two-dimensional eccentricity for the ASC specks was determined to be 1.19, with a median radius of gyration of 321 ± 31 nm median absolute deviation of the median (MAD) (Figures 5I and 5J, STAR Methods and Table 1). The total number of localizations within the speck decreased compared to specks stained with primary antibody and secondary F(ab’)_2_ fragment (Figure 5K). None of the 134 structures observed exhibited a hollow center. DNA-PAINT confirmed our observation of a smaller speck size and also resolved short filamentous extensions at the edge of the structure (Figures 5G, 5H, S8B, and Video S6). The absence of ring-like structures after nanobody labeling is consistent with our hypothesis that the dense regions within the speck limit the accessibility of the labeling probe. The smaller radius of gyration cannot be entirely explained by the high labeling density in the center, and we attribute it to the comparably low binding-affinity of the nanobody (apparent binding constant: 159.5 ± 1.5 nM^26^). Although we are not aware of the binding affinity of the applied ASC antibody, it behaves similarly to typical IgG antibodies with affinities ranging from 10 to 200 pM.^61^ We note that the actual affinity within the speck may potentially also differ from that measured in solution due to steric hindrances or multivalency effects. However, there is a significant affinity difference between antibodies and nanobodies. The nanobody has a single binding site, compared to two for a normal primary IgG antibody, potentially explaining the higher labeling efficiency of the outer, lower-density regions of the speck by the antibody.

**Table 1:** Summary of measurement parameters on unstimulated cells and LPS + Nigericin-treated cells containing an ASC speck

		Unstimulated cells	LPS + Nigericin-treated, Speck-containing cells				
		Primary antibody + secondary AF 647-conjugated F(ab’)_2_ fragment	Primary antibody + secondary AF 647- conjugated F(ab’)_2_ fragment w + w/o additional DL755- conjugated nanobody	Primary antibody + secondary AF 647- conjugated F(ab’)_2_ fragment only	Primary antibody + secondary AF 647- conjugated F(ab’)_2_ fragment w additional DL755- conjugated nanobody	AF 647- conjugated nanobody only	DL 755- conjugated nanobody w additional primary antibody + secondary AF 647- conjugated F(ab’)_2_ fragment staining
# of cells or specks		7	57 (cluster analysis) 60 (speck analysis)	21	39	92	42
# of clusters		2,514	15,566	–	–	–	–
Mean cytoplasmic clusters/area [1/μm^2^]		0.40	0.18	–	–	–	–
Mean # of localizations per cluster		23.19	30.08	–	–	–	–
Median cluster size (2D-*R*_*g*_) [nm]		14.86	20.43	–	–	–	–
Mode cluster size (2D-*R*_*g*_) [nm]		6.04	7.05	–	–	–	–
xy Eccentricity of speck	Mean	–	1.23	1.14	1.29	1.33	1.5
	Median	–	1.19	1.13	1.24	1.23	1.4
	Mode	–	1.02	1.02	1.04	1.07	1.03
	MAD	–	0.08	0.04	0.07	0.11	0.19
2D-*R*_*g*_ of speck [nm]	Mean	–	325.2	332.6	321.2	298.1	158.3
	Median	–	321.3	324.0	319.7	281.9	133.9
	Mode	–	80.4	80.4	242.1	310.6	19.9
	MAD	–	30.9	35.9	29.0	36.5	49.9
Number of localizations per speck	Mean	–	5010	7209	3826	5338	3848
	Median	–	4113	7180	3307	2749	2787
	Mode	–	N/A^a^	1062	N/A^a^	1297	345
	MAD	–	1430	2691	954	1872	2061

a All values occurred just once; MAD: Median absolute deviation of the median; AF 647: Alexa Fluor 647.

Video S6. “Z-stack” super-resolution rendering of an endogenous ASC speck, related to Figure 5 “Z-stack” 3D rendering of an endogenous ASC speck stained with anti-ASC nanobodies conjugated to DNA- PAINT docking strands and measured using DNA-PAINT. Note the overall spherical appearance characterized by a dense core and protruding filaments.

### Two-color super-resolution imaging confirms accessibility differences within endogenous ASC specks

**Figure 6:**
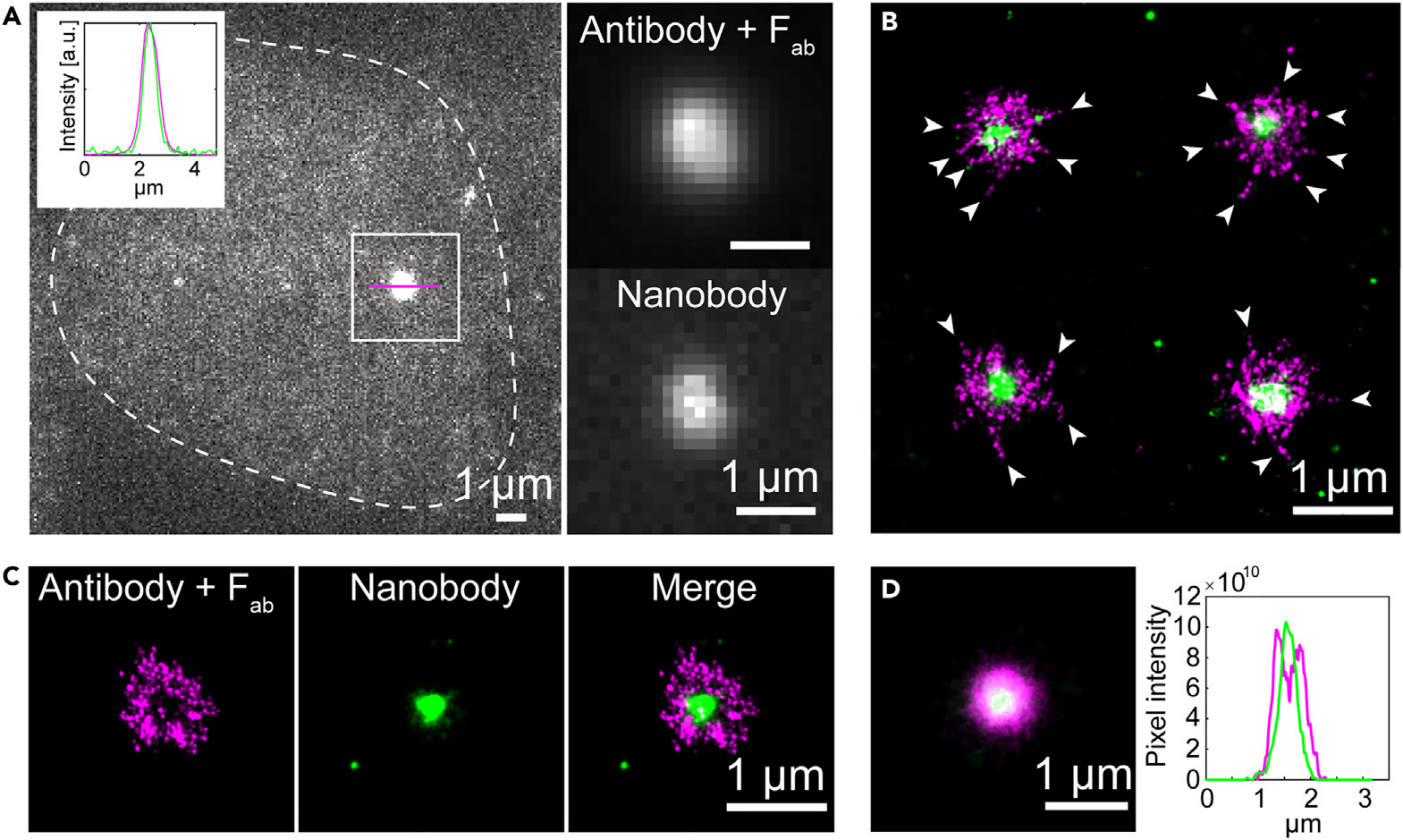
Dual-color dSTORM imaging of ASC specks simultaneously stained with primary antibodies/secondary F(ab’)_2_ fragments and nanobodies against ASC ASC specks were formed in THP-1 caspase-1 knock-out cells after LPS and Nigericin stimulation in the presence of Z-VAD-FMK. (A) A diffraction-limited widefield image of an ASC speck stained with primary antibody and Alexa Fluor 647-conjugated secondary F(ab’)_2_ fragment (the signal of which is shown here) and DyLight 755-conjugated nanobody. The dashed line shows the cell outline. The intensity profile of the ASC speck normalized to the peak of the intensity distribution along a cross-section of the speck (magenta line) is shown in the top left of the image (antibody signal: magenta; nanobody signal: green) showing that both staining approaches stain the same structure. The right part of the panel shows the boxed area split into the signal due to antibody (top) and nanobody (bottom) staining. (B) Four representative dual-color dSTORM reconstructions of ASC specks stained simultaneously with both antibodies and nanobodies (magenta: Alexa Fluor 647; green: DyLight 755). The arrows point toward filaments reaching out from the dense speck core. (C) Dual-color dSTORM reconstruction of a speck showing a ring-like appearance after antibody + F(ab’)_2_-staining (left) labeling, staining of the dense core by the nanobody (center) and the merge of both labeling strategies (right). (D) Alignment of dual-color-labeled specks (n = 35) along the center-of-mass of the antibody + F(ab’)_2_ fragment staining (left) and the intensity profile along a cross-section through the aligned structure (right). Data were obtained on a single cell preparation. See also Figure S10.

To examine whether both the high-density core structure and low-density filaments are present on the same ASC speck, we performed two-color super-resolution microscopy combining both nanobody and antibody labeling on the same sample. Diffraction-limited widefield imaging showed that both labels specifically stained the ASC speck (Figure 6A). Consistent with our previous observation, the signal resulting from nanobody staining was smaller compared to the one obtained from antibody staining. Dual-color dSTORM reconstructions revealed that the antibody-labeled specks have a diameter of about one micrometer in widefield and an *R*_*g*_ of 319.7 ± 29 nm MAD (compared to 324 ± 36 nm MAD under single-labeling conditions, Table 1) with the nanobody staining localized in the center of the speck (Figures 6B and S10). Antibody labeling also resolved filaments and, as observed in the single-color antibody staining, a subset of the ASC specks appeared hollow (Figure 6C). Strikingly, in these particles, the nanobody staining was more compact, with a significantly smaller *R*_*g*_ (134 ± 50 nm MAD, compared to 282 ± 36.5 nm MAD under single labeling conditions, Table 1). We speculate that the higher-affinity antibody outcompetes the nanobody in the lower density regions of the speck, while only the nanobody is small enough to penetrate the dense core of the inflammasome. Aligning the dual-color structures along the center-of-mass of the antibody signals confirmed the observation that nanobody staining is confined to the center of the speck while the antibody complex was found more toward the speck periphery (Figure 6D). Hence, different labeling approaches bring out different features of the ASC speck. While it is typically beneficial to use the smallest available labels,^62^^,^^63^ other factors such as binding affinity and density of the target structure can also play a role in super-resolution imaging.

### ASC forms a scaffold that increases in density with time

From the wealth of information that we gathered using widefield and super-resolution microscopy, we further quantitatively analyzed our data. Since the labeling of ASC with nanobodies was less robust, we limited our analysis to the data collected using primary antibody and secondary F(ab’)_2_ fragment labeling. We first manually segmented the cell and the speck so that we could calculate parameters dependent on the characteristics of the cells and the specks they contained (Figure S2). Widefield images were collected before super-resolution microscopy was performed. Hence, the information from both imaging modalities was available from the same cells. By plotting the total widefield intensity as a function of cell area, a clear correlation was observed (Figure S11A) showing that larger cells express more ASC protein. Similarly, there is a positive correlation between the total number of localizations and cell size (Figure S11B). In fact, the total number of localizations measured using dSTORM correlates well with the total widefield intensity, as one would expect (Figures S11C and S11D). Small corrections for day-to-day variations were performed as discussed in STAR Methods and Figure S12. In contrast, the intensity normalized by the cell area is relatively constant (Figure S11E), suggesting that the concentration of ASC is constant across different cells.

**Figure 7:**
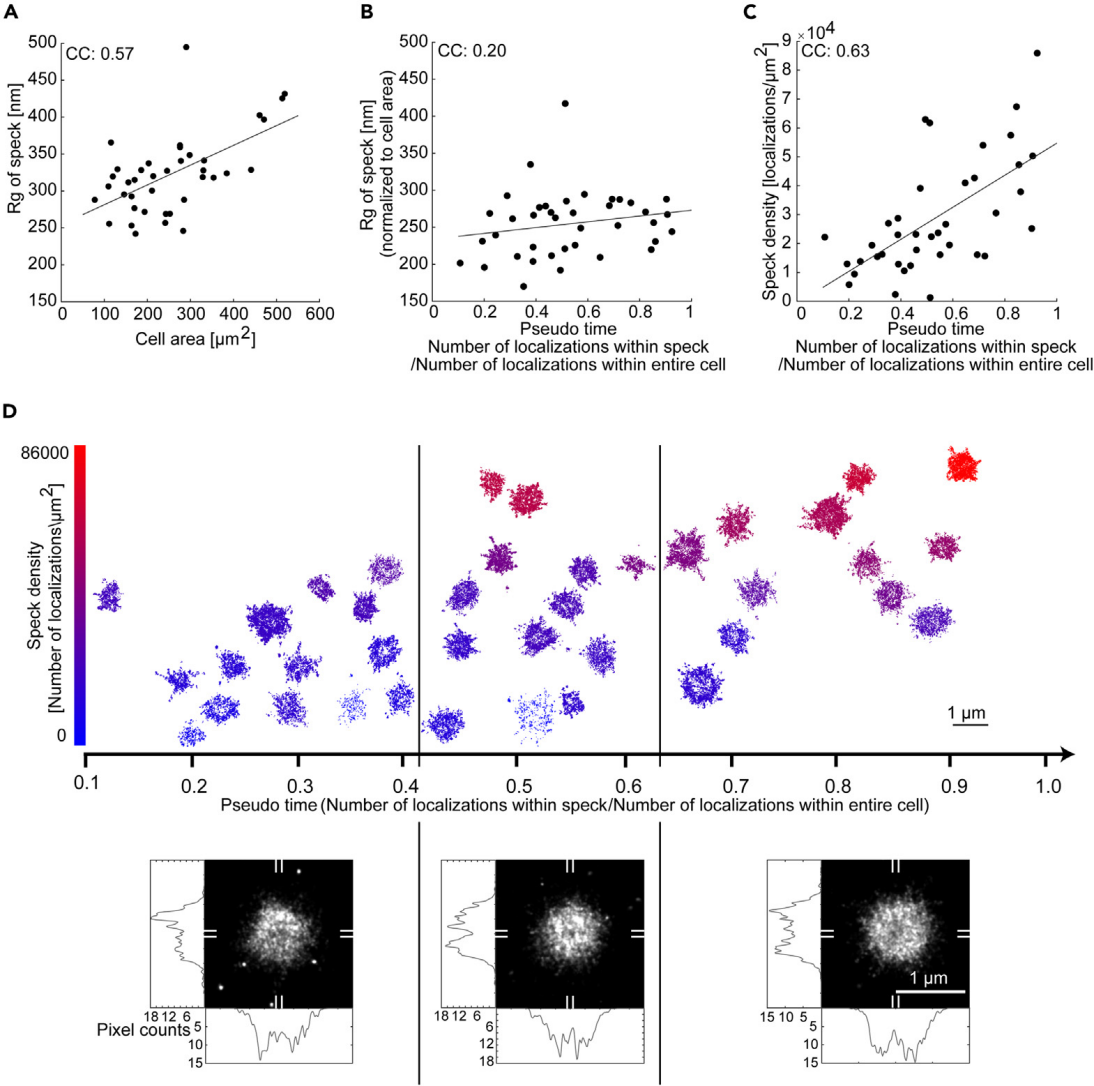
Dynamics of endogenous ASC speck formation (A–C) Scatterplots of speck size (measured as the radius of gyration) as a function of the cell area (A), the speck size, normalized to cell area versus the fraction of the total localizations located within the speck (pseudo time) (B) and the speck density (measured as number of localizations per μm^2^) as a function of pseudo time (C). Larger cells form larger specks and the normalized speck size stays almost constant with increasing pseudo time whereas the speck density increases with pseudo time. CC: Pearson correlation coefficient. (D) Plot of individual speck structures as a function of the pseudo time. The speck density is color-coded (scale bar is shown on the left) and specks are positioned in the vertical direction approximately according to their density. Specks were separated into three time bins (separated by the vertical black lines) and the super-resolution structures aligned and summed (lower panels). Horizontal and vertical cross-sections are shown (determined by averaging along the marked 9 pixel wide regions indicated on the periphery with white lines). The averaged speck structure appears more ring-like at late time points compared to early time points. Data were obtained on three independent cell preparations. See also Figures S2, S11, and S12.

Next, we investigated how the size of the speck varies with cell properties. The size of the speck, determined either by manual segmentation of the speck or via the calculation of the 2D radius of gyration, was found to increase with cell size (Figures 7A and S11F). To investigate how the radius of gyration depends on other parameters, we normalized out the cell-area dependence (Figure S11K see STAR Methods). Interestingly, the radius of gyration only weakly depends on the amount of ASC within the speck (Figures S11L and S11M). The same trend was found when we quantified the speck size manually via the occupied area (Figures S11G–S11I).

Speck assembly is a dynamic process and recruitment of ASC to the perinuclear speck upon NLRP3 inflammasome activation occurs stochastically in the different cells we measured.^18^^,^^47^ Thus, when fixed, different cells represent different stages of the assembly process. We used the ratio of ASC signal in the speck with respect to the total amount of ASC within the entire cell as a metric for the progression of speck assembly, i.e., pseudo time. The pseudo time is only an approximation, which we justify on the basis of live-cell movies^22^^,^^47^ showing the concomitant uptake of ASC in the speck and depletion in the cytosol. For the ASC signal, we utilized the number of localizations as this is more reliable. We observed that the widefield intensity of the speck, as well as the number of localizations within the speck, increased with ASC recruitment along the pseudo time axis, as expected (Figures S11N and S11O). In addition, the cytosolic, non-speck ASC signal consistently decreased with the pseudo time regardless of whether we quantified it using the number of localizations, the cluster density or the localization density (Figures S11R–S11T). We did not observe a clear decrease in the integrated widefield intensity excluding signal from the ASC speck with the pseudo time as would have been expected (Figure S11Q). We attribute this to the low level of protein remaining in the cytosol upon ASC recruitment to the speck, and the autofluorescence signal contributing a significant fraction of the entire signal. A plot of the radius of gyration versus pseudo time showed little change in the size of the speck with pseudo time (Figure S11U). This also holds true for the speck area (Figure S11V), but the observation that the speck size depends on cell area could potentially confound the trend. Hence, we examined the normalized speck size as a function of pseudo time. The normalized radius of gyration of the speck (corrected for the correlation with cell area) increases only slightly during the course of ASC recruitment (Figure 7B). Similarly, when we normalized out the increase in speck area with cell size, only a small increase with pseudo time is observed (Figure S11P). We also plotted cell area with pseudo time (Figure S11W), and observed no correlation. Although this was expected, it also verifies that the endogenous ASC speck formation is complete before pyroptosis and cell shrinkage is triggered. If the radius of gyration of the speck does not depend on pseudo time but the ASC content of the speck does, then we would expect the ASC density in the speck to increase with time. We investigated the speck density (localizations per area) and found it to be largely independent of the cell area (Figure S11X) but clearly increasing with ASC recruitment (Figure 7C). In line with this observation, there is a clear correlation between the speck density and the amount of ASC within the speck, calculated either via the integrated widefield intensity within the speck (Figure S11Y) or the number of localizations within the speck (Figure S11Z). Hence, we conclude that the density and, to a much lesser extent, the size of the speck increases with ASC recruitment.

To visualize potential structural rearrangements of the speck, we plotted the endogenous ASC specks as a function of pseudo time (Figure 7D). No clear structural progression was visible. To look for more subtle changes during ASC recruitment, we divided specks into three groups according to the fraction of recruited ASC. We aligned the complexes in each group to their center-of-mass. The resulting sum projection showed an increased tendency to form a ring-like structure at later stages of recruitment, which is consistent with the observation that the specks become denser with time and thereby exclude the antibody from the center of the speck (Figure 7D lower panel).

**Figure 8:**
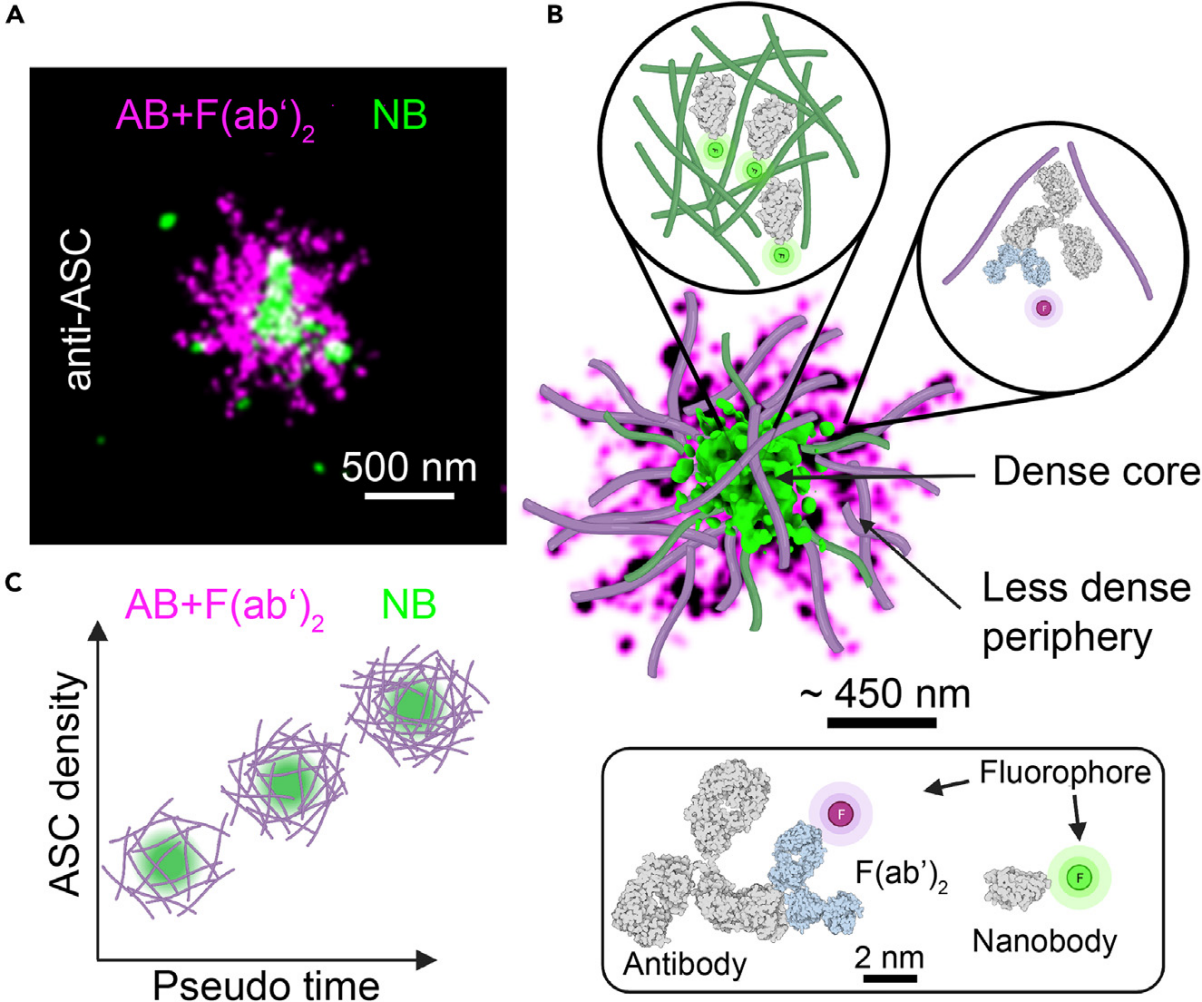
Model of endogenous ASC speck formation (A) 2-color dSTORM reconstruction of an ASC speck stained with primary anti-ASC antibody and secondary Alexa Fluor 647-conjugated F(ab’)_2_ fragment (magenta) and a DyLight 755-conjugated anti-ASC nanobody (green). The nanobody signal is concentrated in the center of the structure whereas the primary antibody/secondary F(ab’)_2_ fragment staining is also observed in the periphery of the structure. (B) Schematic model for the supramolecular structure of the ASC speck based on measured data overlaid with modeled filaments. The structure is characterized by a dense core and a less dense periphery. The zoom ins illustrate that the nanobody is able to penetrate into the dense core of the speck whereas the antibody preferably labels the less dense periphery of the structure. The insert at the bottom of (B) illustrates the size difference between the labeling probes. (C) Illustration of speck formation over time. The ASC speck forms from intertwined filaments assembling into a scaffold whose size scales with the cell size. Early stages of speck formation are characterized by a loose assembly into which the antibody (magenta) and nanobody (green) can penetrate. Further ASC recruitment into the speck leads to denser structures but only a marginal increase in its size. Antibody and F(ab’)_2_ fragment staining is sterically excluded from the dense center of the structure resulting in an overall ring-like appearance. The smaller nanobody stains the dense core of the speck but is washed away from the less dense regions of the structure due to its lower binding affinity. AB, antibody; F(ab’)_2_, F(ab’)_2_ fragment; NB, nanobody.

Taken together, our data suggest that the speck forms initially as a loose scaffold of intertwined filaments whose size depends on the cell area and which becomes denser, but not much larger over time by recruiting and incorporating more ASC (schematically shown in Figure 8).

## Discussion

The supramolecular NLRP3 inflammasome complex is a central component of the innate immune system, driving the maturation of the proinflammatory cytokines IL-1β and IL-18 as well as pyroptosis, a proinflammatory form of cell death. The protein ASC is critical during inflammasome formation and, upon cell stimulation, is recruited into a single, condensed structure, the ASC speck. ASC speck formation and organization are difficult to study due to the small size and heterogeneity of the complex. We used a combination treatment of LPS and Nigericin, which led to robust induction of ASC specks with a broad distribution in ASC content (Figure 1). Other triggers of NLRP3 inflammasome activation are known, which could potentially lead to ASC speck formation via a different mechanism and thereby result in a different structure of the speck. Here, we chose LPS and Nigericin as it is the standard protocol used in the field of NLRP3 activation.^43^ To circumvent caspase-1-driven cell death and thereby increase the number of fixed, speck-containing cells, we used caspase-1 knock-out cells for all described experiments (except for the measurements on BlaER1 cells). We cannot completely exclude that the absence of caspase-1 has an effect on the observed ASC speck structure.

In diffraction limited widefield microscopy, the complexes appear as spherical structures with very few morphological features. We then used dSTORM and DNA-PAINT, super-resolution techniques, to investigate the organization of endogenous ASC in the speck as well as in the cytoplasm. dSTORM measurements were performed on a total of 358 cells and 251 specks were analyzed in detail. This allowed us to reconcile several controversies regarding the reported structures of the ASC speck.

Our cluster analysis of the non-speck bound cytosolic ASC fraction revealed that the size of the vast majority of ASC oligomers remained unchanged between unstimulated and speck-containing cells (Figures 2E and 2F). Considering the size of the labels, we measured an average *R*_*g*_ in unstimulated cells of ∼15 nm (Figure 2F). This is in good agreement with an atomic force microscopy (AFM) study that measured the dimensions of full-length human ASC assemblies, which organized into disk-like oligomers of 1 nm in height and ∼12 nm in diameter *in vitro.*^11^ Upon stimulation, we observe a small increase in the number of larger clusters in the cytosol. This could be due to preassembly of a portion of ASC into higher-order ASC oligomers that later associate into the speck as has been suggested in the literature.^8^^,^^64^ Western blot analysis of inflammasome activated cells found different levels of ASC multimerization in addition to the ASC speck with a large proportion of the protein being dimeric.^35^

In our systematic measurements of ASC speck formation, the cells either showed a single speck or the ASC protein appeared homogenously distributed throughout the cell. We did not observe any concentration dependence of ASC as a function of distance from the speck (e.g., Figure 2C), consistent with previous observations^47^ made in cells overexpressing ASC. This is understandable since the entire pool of ASC is recruited into the ASC speck within a few minutes during complex formation.^8^^,^^18^^,^^47^^,^^65^ We were still able to detect cytosolic ASC in speck-containing cells. The percentage of ASC recruitment we observed was typically 20% or above (with one exception) suggesting that the initial recruitment of ASC is faster than the fixation processes. This is consistent with live-cell imaging measurements in where the increase in speck size occurred over a time interval of ∼100 s.^47^ Our pseudo-time observations indicate that it is mostly the speck density but not the speck size that increases with the percentage of ASC being recruited during the later stages of assembly.

The specks we recorded were smaller in size than those measured previously upon overexpression of ASC.^22^^,^^25^ This is in line with our observation that the radius of gyration increases with total ASC content (Figure 7A). NLRP3 inflammasome formation has recently been suggested to occur at the MTOC^44^^,^^59^ and a correlation has been observed between the size of the MTOC and the cell size (at least in *C*. *elegans*).^66^ Our measurements clearly showed no colocalization between the MTOC and the ASC speck. In contrast to Magupalli et al.^44^ who measured after 30 min of Nigericin stimulation, our experiments were performed at 90 min. Also Yang et al.^67^ mention colocalization between the MTOC and ASC speck in bone marrow-derived macrophages (BMDMs) with the highest probability for colocalization (∼40%) occurring at 20 min after Nigericin stimulation. They also speculate that the ASC speck, after assembly at the MTOC, then migrates into the cytosol.^68^ A recent preprint also postulates that inflammasome formation after Nigericin stimulation can occur at the MTOC as well as free in the cytoplasm with the MTOC-dependent formation being more prevalent at early time points (25 min) after stimulation.^69^ Furthermore, since the microtubule network is almost completely disassembled 90 min after Nigericin stimulation, we cannot exclude that the lack of colocalization we observe is due to the loss of microtubule-mediated tethering of the speck to the MTOC. However, in individual cells, formation of the ASC speck begins stochastically after addition of the stimulus. The physiologically relevant time point is the initiation of processes leading to speck formation in the individual cells rather than the time point of stimulus addition. In our study, we represent the dynamics of ASC speck formation via the pseudo time. As we observe a large range of pseudo times during our experiments (Figure 7), it is unlikely that the difference in time points after the addition of the Nigericin stimulus is responsible for the lack of colocalization between the ASC speck and the MTOC. However, this argument does not hold if disassembly of the microtubule network is initiated by a different trigger than ASC speck formation. Hence, future studies should investigate earlier time points after Nigericin stimulation.

To reliably quantify the attributes of the amorphous, heterogeneous speck, we combined the results from 251 specks. The endogenous ASC speck has a size variation, determined from the 2D radius of gyration of antibody-stained specks, ranging from 250 to 500 nm with an average radius of gyration of 321 ± 31 nm MAD (Figure 5J). The radius of gyration of the ASC speck corresponds to the previously published value of ∼600 nm diameter for the endogenous structure (measured from cross-sections through the speck).^35^ Typically, one would expect the size of the speck to increase during assembly and hence the distribution of sizes to depend, in part, on the stage of assembly at which they are measured. However, when calculating the amount of ASC located within the speck relative to the total amount of ASC within the cell (i.e., our pseudo time), we found that the speck size depends only to a minor degree on the amount of recruited ASC (Figure 7B).

We found the majority of specks appeared as amorphous objects with an overall spherical structure, as determined from the calculated eccentricity (Figure 5I). These results are consistent with previous observations.^8^^,^^29^ In addition, we observed that some specks exhibit filamentous extensions protruding from the dense core. Although several studies suggested that the ASC speck is made up of intertwined filaments,^20^^,^^22^^,^^26^^,^^27^ this has not yet been confirmed for the endogenous, unperturbed structure inside cells. We observe a dense core with filaments protruding from the endogenous ASC speck, which would be consistent with this hypothesis.

Although we could not analyze the observed filaments in detail due to the limited labeling density, we did observe filaments of varying length and thickness. Some specks exhibit many short and faint fibrils (Figures 5E–5H) while others show a small number of longer filaments protruding from the edge of the speck core (Figure 3C). The thin fibrils are reminiscent of the fibrils resolved by EM at the edge of the *in vitro* formed ASC assembly.^22^ A similar variation in thickness of the filaments protruding from the dense speck core has been observed in immortalized ASC^−/−^ BMDMs transduced with ASC tagged using mCherry.^20^ Interestingly, EM data of *in vitro* assembled structure from full-length human ASC protein suggested that individual ASC filaments can laterally stack via the exposed CARD domains and the authors raise the question of whether this could also happen in the endogenous structure.^17^ The average filament thickness we obtained on the aforementioned filaments agrees with the thickness reported for a single filament (∼16 nm)^17^ suggesting that, for most filaments, lateral stacking does not take place.

It is important to mention that the majority of our experiments were conducted in caspase-1 knock-out cells. Hence, we cannot exclude that the lack of caspase-1 binding to ASC has an influence on the appearance of the speck. Experiments performed using DNA-PAINT on BlaER1 cells that express caspase-1 showed the same dense, amorphous core with protruding filaments. This strongly suggests that the measured structures are indicative of the morphology of the ASC speck and is consistent with what has been observed in experiments in cells with ASC overexpression.^20^^,^^22^^,^^25^ However, in comparison to the previous observations of the speck formed after overexpression,^22^^,^^25^ we found fewer and shorter filaments protruding from the endogenous ASC speck core with the majority of filaments being 500 nm long or shorter. It is conceivable that overexpression leads to a larger number of filaments emanating from the formed speck. It is also possible that caspase-1 influences the filament length as our measurements in BlaER1 cells that express caspase-1 showed longer filaments than the ones observed in THP-1 caspase-1 knock-out cells (Figures 3C and S4B). The longer filament lengths are in line with a previous report where *in vitro* experiments measured filament lengths of 500–2000 nm for full length mouse ASC.^16^ However, we cannot entirely rule out that the differences in filament length are due the details of the super-resolution approach used.

An overall ring-like appearance of the ASC speck has been proposed since its discovery.^3^ However, the question of how the ring-like assembly and the irregular structure made up of intertwined filaments relate to each other remains unanswered.^39^^,^^40^^,^^41^^,^^42^ Here, we provide new data that offers an explanation for the observed ring-like structure. Complementing previous studies, which showed a ring-like assembly of ASC following antibody labeling, we used labels of different sizes, primary antibodies labeled with secondary F(ab’)_2_ fragments and nanobodies. While primary antibody plus secondary F(ab’)_2_ fragment labeling occasionally showed ring-like structures, simultaneous labeling with nanobodies revealed a dense core in the speck (Figure 6). Hence, we conclude that the endogenous speck can be divided into two different regions: a dense core and a less dense periphery (Figure 8). This would imply that the ring shape originates from the primary and/or secondary antibody being less likely to penetrate into the dense center of the structure and thus leading to a decrease in labeling efficiency at the center of the structure. Conversely, the less dense structures at the periphery are less efficiently labeled by the nanobody as it has only one binding site per protein with, in this case, a relatively low binding affinity (∼160 nM). This also demonstrates the importance of verifying super-resolution structures using different labeling strategies and approaches.

Taken together, we investigated the nanoscale organization of the endogenous ASC speck using a variety of fluorescence microscopy approaches including super-resolution microscopy. We found that the speck size was heterogeneous and correlated with cell size. The speck contained a dense core with filaments protruding from the center of the speck. This was observed for dSTORM and DNA-PAINT measurements in THP-1 cells as well as DNA-PAINT measurements in BlaER1 cells. These results are consistent with the model of the ASC speck being an assembly of intertwined filaments. We found that ASC speck assembly does not occur at the MTOC under the conditions of our experiment. By using differently sized labels, we found that the ring-like appearance of the speck is a result of the labeling approach and limited access of the primary antibody and/or F(ab’)_2_ fragment to the dense core of the speck. Conversely, the small nanobody was able to label the center of the speck but was less efficient in labeling the low-density periphery regions due to the lower affinity of this nanobody (Figure 6). Hence, it is important to verify super-resolution structures using different labeling approaches. Finally, by measuring the fraction of recruited ASC, we hypothesize that speck formation starts with a loose scaffold that becomes denser but only marginally larger during ASC speck formation.

### Limitations of the study

Our analysis is limited to the single time point of 90 min after Nigericin stimulation. This time point provides a high probability of detecting ASC specks but is significantly longer than the 25–30 min in studies that detected colocalization between ASC specks and the MTOC.

## STAR★Methods

### Key resources table

**Table:** 

REAGENT or RESOURCE	SOURCE	IDENTIFIER
**Antibodies**		

mouse anti-human ASC antibodies (TMS-1)	Biolegend	Cat# clone HASC-71
F(ab’)_2_-goat anti-mouse IgG (H + L) cross-adsorbed Alexa Fluor 647-conjugated	Thermo Fisher Scientific	Cat# A-21237; RRID: AB_2535806
polyclonal rabbit anti-pericentrin	Abcam	Cat# ab4448; RRID: AB_304461
goat anti-rabbit IgG (H + L) highly cross-adsorbed secondary antibody Alexa Fluor Plus 488	Thermo Fisher Scientific	Cat# AB-2633280
donkey anti-mouse IgG (H + L) highly cross-adsorbed secondary antibody Alexa Fluor 555	Thermo Fisher Scientific	Cat# A-31570; RRID: AB_2536180
rabbit β-tubulin antibody	Cell Signaling	Cat# 5346; RRID: AB_1950376
anti-rabbit polyclonal antibody (3xR3)	Massive Photonics	Gift
donkey anti-mouse antibody	Jackson ImmunoResearch	Cat# 715-005-151; RRID: AB_2340759
F(ab’)_2_-goat anti-mouse IgG (H + L) cross-adsorbed Alexa Fluor 647-conjugated	Thermo Fisher Scientific	Cat# A-21237; RRID: AB_2535806

**Chemicals, Peptides, and Recombinant Proteins**		

Roswell Park Memorial Institute 1640 medium	Thermo Fisher Scientific	Cat# 21875034
Fetal Bovine Serum	Thermo Fisher Scientific	Cat# 10500064
sodium pyruvate	Thermo Fisher Scientific	Cat# 11360039
Penicillin/Streptomycin	Thermo Fisher Scientific	Cat# 15140122
Phorbol 12-myristate 13-acetate (PMA)	Enzo Life Sciences	Cat# BML-PE160-0001
Lipopolysaccharide (LPS) from *E. coli* K12 cells	Invivogen	Cat# tlrl-peklps
pan-caspase inhibitor Z-VAD	Invivogen	Cat# tlrl-vad
Dimethylsulfoxid	Carl Roth	Cat# A994.1
Nigericin	Sigma-Aldrich	Cat# N7143-5MG
Ethanol	VWR	Cat# 20821330
paraformaldehyde	Electron Microscopy Sciences	Cat# E15710-S
normal goat serum	Thermo Fisher Scientific	Cat# 16201
Triton X-100	Sigma-Aldrich	Cat# T8787
NH_4_Cl	Sigma-Aldrich	Cat# 254134
Saponin	Sigma-Aldrich	Cat# 47036
NaN_3_	Sigma-Aldrich	Cat# S2002
BSA	Sigma-Aldrich	Cat# A7030
Image-iTFX Signal Enhancer	Thermo Fisher Scientific	Cat# R37107
IL-3	Peprotech	Cat# 200-03 B
M-CSF	Peprotech	Cat# 300-25 B
b-Estradiol	Sigma-Aldrich	Cat# E8875
EDTA	Thermo Fisher Scientific	Cat# AM9260G
PCA	Sigma-Aldrich	Cat# 37580
PCD	Sigma-Aldrich	Cat# P8279

**Deposited data**		

Imaging data of the study	Zenodo	https://doi.org/10.5281/zenodo.8407512

**Software and algorithms**		

MATLAB	Mathworks	2018b
Custom MATLAB scripts for data analysis	Mathworks	https://gitlab.com/lamb_lab_software/analysis-of-smlm-localizations
ImageJ	Schneider et al.^70^	https://imagej.net/
Micromanager	Edelstein et al.^71^^,^^72^	https://micro-manager.org/

### Resource availability

#### Lead contact

Further information and requests for resources and reagents should be directed to and will be fulfilled by the lead contact, Don Carroll Lamb (d.lamb@lmu.de).

#### Materials availability

• THP-1 ASC KO, THP-1 Caspase-1 KO cells and BlaER1 ASC-TagRFP cells were not generated for this manuscript but can be requested from Veit Hornung.
• The plasmid encoding for the ASC nanobody was provided by Prof. Dario Alessi, University of Dundee via the MRC – Protein Phosphorylation and Ubiquitylation Unit [DU 54832]. We cloned the ASC nanobody sequence into the pCoofy2 expression plasmid.^73^

#### Data and code availability

• Data:
  The localization data has been deposited at Zenodo and are publicly available as of the date of publication. DOIs are listed in the key resources table.
• Code:
  All original code has been deposited at Gitlab and is publicly available as of the date of publication. DOIs are listed in the key resources table.
• All other items:
  Any additional information required to reanalyze the data reported in this paper is available from the lead contact upon request.

### Experimental model and study participant details

If not stated otherwise, all reagents were purchased from Thermo Fisher Scientific, Massachusetts, USA.

#### Cell lines

##### THP-1 cells

THP-1 cells were cultivated at 37°C, 5% CO_2_ in Roswell Park Memorial Institute 1640 medium (21875034) supplemented with 10% (v/v) heat-inactivated fetal bovine serum (FBS) (10500064), 1 mM sodium pyruvate (11360039) and 100 U/mL Penicillin/Streptomycin (15140122), and maintained at a density between 1x10^5^ and 1x10^6^ cells per milliliter. To maximize the number of cells showing an ASC speck in our study, we used Caspase-1 knockout THP-1 cells additionally treated with a pan-caspase inhibitor (Z-VAD-FMK) as Caspase activation upon inflammasome activation leads to cell death. To control for unspecific staining we made use of THP-1 ASC KO cells. THP-1 cells are a monocytic cell line derived from a male human patient with acute monocytic leukemia. THP-1 cells were provided by the lab of Veit Hornung but have not been additionally authenticated.

##### BlaER1 cells

BlaER1 ASC-TagRFP cells were cultivated as described for the THP-1 cells. The cells were provided by the lab of Veit Hornung. They were not additionally authenticated. BlaER1 cells were derived from the RCH-ACV cell line, originating from a female human with B-Lymphoblastic Leukemia/Lymphoma. During the course of this study, we were informed that the BLaER1 cells contain the Squirrel Monkey Retrovirus proviral genome.

### Method details

#### Seeding and differentiation of THP-1 cells and activation of the NLRP3 inflammasome in THP-1 cells

One day prior to seeding the cells, either coverslips (1.5, Menzel Gläser, 18 mm) or Ibidi μ-Slide 8-well high glass bottom slides (80807, Ibidi, Gräfelfing, Germany) were coated with 0.01% poly-*l*-ornithine solution (A-004-C, Merck-Millipore, Massachusetts, USA) in the dark. Cells were seeded at a density of ∼75×10^3^/cm^2^ in culture medium supplemented with 162 nM (100 ng/mL) Phorbol 12-myristate 13-acetate (PMA) (BML-PE160-0001, Enzo Life Sciences, Lörrach, Germany) and differentiated into macrophage-like cells for three days. To increase NLRP3 protein expression, cells were primed for 3 h with 1 μg/mL Lipopolysaccharide (LPS) from *E. coli* K12 cells (Ultrapure tlrl-peklps, Invivogen, San Diego, California, USA) dissolved according to the manufacturer’s protocol. To suppress cell death due to caspase activity, cells were subsequently treated with 20 μM of the pan-caspase inhibitor Z-VAD-FMK (tlrl-vad, Invivogen) dissolved in Dimethylsulfoxid (A994.1, Carl Roth, Karlsruhe, Germany) for 60 min. The NLRP3 inflammasome response was activated by incubating the cells with 10 μM Nigericin (N7143-5MG, Sigma Aldrich, Missouri, USA) dissolved in Ethanol (20821330, VWR, Ismaning, Germany) for 90 min.

#### ASC immunofluorescence of THP-1 cells with primary antibody and secondary F(ab’)_2_ fragment and for double-labeling with an ASC nanobody

All steps were performed at room temperature if not stated differently. Cells were washed once with phosphate-buffered saline (PBS) solution to remove remaining serum proteins, fixed for 15 min in the dark with 4% paraformaldehyde (PFA) (E15710-S, Electron Microscopy Sciences, Pennsylvania, USA) in PBS. Subsequently, PFA was rinsed once with PBS and then quenched by rinsing once with 100 mM NH_4_Cl (254134, Sigma-Aldrich) in PBS followed by a 15 min incubation with 100 mM NH_4_Cl in PBS. Permeabilization and blocking was done for 30 min in 10% normal goat serum (NGS) (16201), 0.5% Triton X-100 (T8787, Sigma-Aldrich) in PBS followed by a washing step with PBS and 30 min incubation in Image-iTFX Signal enhancer (R37107). After two additional PBS washing steps, cells were incubated overnight at 4°C with purified monoclonal mouse anti-human ASC antibodies (TMS-1) (clone HASC-71) (Biolegend, California, USA) at a final concentration of 67 nM (10 μg/mL) diluted in 10% NGS, 0.5% Triton X-100 in PBS. 25 μL of this solution was applied to the fixed cells on a coverslip. The plasmid encoding for the ASC nanobody was provided by Prof. Dario Alessi, University of Dundee via the MRC – Protein Phosphorylation and Ubiquitylation Unit [DU 54832]. The nanobody encoding sequence with a C-terminal cysteine was inserted into the pCoofy2 vector^73^ and amplified for labeling and expressed at the protein production core facility of the Max-Planck-Institute for Biochemistry in Martinsried, Germany. Parts of the nanobody were conjugated to Alexa Fluor 647 in house as described in^74^ and at Nanotag Biotechnologies GmbH, Göttingen, Germany. In the case of dual-color labeling, DyLight755-conjugated anti-ASC nanobody was purchased from Nanotag and mixed into the antibody solution at 82 nM (1 μg/mL) final concentration. Non-specific sticking of antibody and nanobody was minimized by washing three times with 0.1% Triton X-100 in PBS. Labeling with secondary F(ab’)_2_-goat anti-mouse IgG (H + L) cross-adsorbed Alexa Fluor 647-conjugated F(ab’)_2_ fragment (A-21237) was performed at 200 ng/mL final concentration in 10% NGS, 0.5% Triton X-100 in PBS for 1 h followed by three washing steps with 0.1% Triton X-100 in PBS and postfixation with 3% PFA in PBS for 10 min. The specificity of the staining procedure was confirmed by staining THP-1 caspase-1 knock-out cells only with the F(ab’)_2_ fragment without previous administration of the primary antibody as well as by applying the staining protocol to THP-1 ASC knock-out cells (Figure S13).

#### ASC immunofluorescence of THP-1 cells with ASC nanobody

THP-1 caspase-1 knock-out cells were activated for the NLRP3 inflammasome, fixed and quenched as described above followed by permeabilization with 0.05% Saponin (47036, Sigma-Aldrich), 1% BSA (A7030, Sigma-Aldrich), and 0.05% NaN_3_ (S2002, Sigma-Aldrich) in PBS for 20 min. Afterward, the sample was washed for 2 min with PBS and then the sample was blocked for 30 min with Image-iTFX Signal Enhancer (R37107). The sample was then washed twice for 2 min with PBS and stained against ASC with 1 μg/mL Alexa Fluor 647-conjugated nanobody and postfixed for 10 min in 3% PFA in PBS. The specificity of the staining was confirmed using THP-1 ASC knock-out cells (Figure S14).

#### ASC and pericentrin immunofluorescence of THP-1 caspase-1 knock-out cells

For the immunofluorescence staining against ASC and pericentrin (Figure 4), purified polyclonal rabbit anti-pericentrin (ab4448, Abcam, Cambridge, UK) antibodies were used together with the monoclonal mouse anti-human ASC (TMS-1) (clone HASC-71) (Biolegend, California, USA) antibodies at a concentration of 67 nM (10 μg/mL). The antibodies were labeled with goat anti-rabbit IgG (H + L) highly cross-adsorbed secondary antibody Alexa Fluor Plus 488 (AB_2633280) and donkey anti-mouse IgG (H + L) highly cross-adsorbed secondary antibody Alexa Fluor 555 (A-31570). Cells were kept in fresh PBS during observation.

#### Confocal imaging

For the confocal imaging shown in Figure 1, the samples were embedded in ProLong Gold (P10144) on standard glass slides for at least one day before imaging. Microscopy was then performed on a Leica SP8 STED 3x equipped with a 470–670 nm white light laser and a 100 x PlanApo/NA 1.4 objective. The spinning disk confocal microscopy images shown in Figure S13A were measured on a Zeiss Cell Observer Spinning Disk microscope using a 405/488/568/647 nm polychroic mirror. Alexa Fluor 647 and DAPI were excited using a 639 nm and 405 nm laser, respectively. Fluorescence was separated using a 660 nm shortpass filter and recorded on the two Evolve 512 electron-multiplying charge-coupled device cameras (Photometrics) of the system equipped with a 525/50 and a 690/50 bandpass filter, respectively.

For the confocal imaging shown in Figure 4, the channels corresponding to the Alexa Fluor 488 (Pericentrin) and Alexa Fluor 555 (ASC) were acquired on the above-mentioned Zeiss Cell Observer Spinning Disk microscope. For excitation, the 488 nm and the 561 nm lasers were used alternately and focused on the field-of-view using the same 405/488/568/647 nm polychroic mirror. Fluorescence was recorded on the same Evolve 512 camera of the setup using the 660 nm shortpass filter combined with a 525/50 or a 629/62 bandpass filter for Alexa Fluor 488 and Alexa Fluor 555 channels respectively. Z-stacks were acquired using Nyquist-optimized sampling and a maximal intensity projection was performed using ImageJ.^70^ For cell counting, multiple regions of 5x5 field-of-views were acquired using the tile function of the Zeiss Zen Blue software.

#### dSTORM imaging and analysis

Samples were imaged on a flat field-optimized widefield setup.^52^ Briefly, lasers of 405 nm, 642 nm and 750 nm were expanded and reflected into a 60× objective (CFI60 PlanApo Lambda 60x/NA 1.4, Nikon) using a custom appropriate dichroic mirror (ZT405/561/642/750/850rpc, Chroma, Vermont, USA). Fluorescence emission was imaged onto a sCMOS camera (Prime, Teledyne Photometrics, Tucson, USA) using one of two bandpass filters (ET700/75M and ET810/90m, Chroma) combined with a shortpass filter (FF01-842/SP, Semrock, New York, USA) and a tube lens of 200 mm focal length. The microscope was controlled using Micromanager.^71^^,^^72^ Widefield images were collected prior to dSTORM recordings. We typically recorded between 20,000 and 80,000 frames at 10 ms exposure time. For 3D dSTORM, we introduced a cylindrical lens (f = 1000 mm, LJ1516RM-A, Thorlabs, New Jersey, USA) into the emission path. Single- and dual-color single-molecule localization microscopy (SMLM) imaging was carried out with an optimized SMLM buffer^75^ containing 10 mM Tris-HCl, 10 mM cysteamine, 50 mM 2-mercaptoethanol, 2 mM cycelooctatetraen, 2.5 mM protocatechuic acid and 125 nM protocatechuate dioxygenase. Single molecules were localized using an sCMOS-specific localizer routine introduced by Huang et al.^76^ and included in a custom MATLAB program used for data analysis. To exclude non-specific localizations, filter parameters were adjusted using datasets acquired using ASC knock-out THP-1 cells. Drift correction was performed using the Redundancy Cross-Correlation (RCC) algorithm introduced by Wang et al.^77^ The cells and specks were manually segmented. Cytoplasmic localizations were clustered using DBSCAN where a cluster was defined as a group of 10–300 localizations within a search radius of 70 nm^49^ or Ripley’s K function.^50^ Super-resolution images were reconstructed using ThunderStorm^78^ with 10× magnification and applying a Gaussian blur of one pixel (10.6 nm). For dual-color datasets, the localizations in the DyLight 755 channel were registered to the ones in the Alexa Fluor 647 channel by an affine transformation calculated from widefield images of immobilized TetraSpeck Microspheres (T7279) on a plasma cleaned coverslip recorded in both channels. 3D representations were rendered using Chimera X.^79^

The eccentricity, the radius of gyration and the number of localizations of the specks were calculated from the manually segmented localizations within the structure. The radius of gyration was calculated as the square root of the sum of the variances of the x and the y coordinates of the individual localizations within each speck. As, in most cases, the specks are amorphous, spherical structures and we have significantly better resolution in the radial dimension, we limited the calculated radius of gyration to two-dimensions. The eccentricity of the individual specks was determined from the localizations by first calculating the covariance matrix of the individual localizations. From the covariance matrix, the eigenvectors were calculated, which provide a measure for how elliptical the distribution of localizations is and calculates the direction of the major and minor axes of the ellipse. We then take the square root of eigenvectors, which give a measure of length for the different axes. Finally, the eccentricity is calculated by taking the ratio of the major axis (square root of the maximum eigenvector) to the minor axis (square root of the minimum eigenvector). Circular objects have an eccentricity near 1. For our microscope we determined an experimental localization precision of σ_xy_ = 12 nm for Alexa Fluor 647 and σ_xy_ = 21 nm for DyLight755.^52^

For the quantitative image analysis, measurements of the integrated widefield intensity were corrected by subtracting background counts determined individually for each field of view by averaging pixel counts in an area absent of cellular signal. To calibrate for the experimental differences observed between measurement days, the widefield intensity per cell area and the number of detected localizations per widefield intensity were corrected separately for each measurement day. For this, we used the correlation of fluorescence intensity with cell size and the number of localizations versus widefield intensity. The correlation was determined for each day by fitting the data with a line and the slope was scaled to correspond to the maximum of all experiment days. The scaled widefield intensity and number of localizations were then used for the ensuing analyses. Figure S11 illustrates the applied correction procedure.

The cell area dependence of the radius of gyration (*R*_*g*_) was normalized out by first plotting the *R*_*g*_ against the cell area and fitting the data with a line. Subsequently for each data point, the product of the slope of the line and the value of the cell area was calculated and subtracted from the *R*_*g*_ value. The speck area was normalized analogously.

#### BlaER1 cell preparation and DNA-PAINT imaging

Cells were transdifferentiated for 7 days at 60,000 cells/cm^2^ in medium containing 3.4 nM (500 UI/mL, 50 ng/mL) IL-3 (200-03 B, Peprotech, New Jersey, USA), 1.36 nM (50 UI/mL, 50 ng/mL) M-CSF (300-25 B, Peprotech) and 500 nM β-Estradiol (E8875, Sigma-Aldrich) in cell culture-treated plastic bottom slides (80826, Ibidi). Cells were then trypsinized, transferred to glass bottom slides coated with poly-*l*-ornithine as described for THP-1 cells in cultivation medium without growth factors at the same density and incubated overnight at 37°C, 5% CO_2_. The next day, the NLRP3 inflammasome was activated by priming the cells for 14 h with 200 ng/mL Lipopolysaccharide (LPS) from *E. coli* K12 cells in medium followed by caspase inhibition with 20 μM Z-VAD-FMK incubation for 60 min as described for THP-1 cells and 3 h incubation with 6.5 μM Nigericin. Cells were fixed and stained as described for THP-1 cells with the exception that a secondary donkey anti-mouse antibody (715-005-151, Jackson ImmunoResearch, Philadelphia, USA) conjugated to a P3 docking strand (TTCTTCATTA) was used instead of the F(ab’)_2_ fragment. Specks were identified by excitation of TagRFP with a 561 nm laser, followed by photobleaching of the TagRFP signal and DNA-PAINT data were recorded using a P3-Cy3B imager strand (AATGAAGA-Cy3B) at 1 nM concentration in imaging buffer (1× PBS, 1 mM EDTA (AM9260G) and 500 mM NaCl (AM9759), pH 7.4; supplemented with 1× trolox (238813, Sigma-Aldrich), 1× PCA (37580, Sigma-Aldrich) and 1× PCD (P8279-25UN, Sigma-Aldrich) and HiLo illumination at 1.2 kW/cm^2^ excitation with the same laser at 100 ms camera exposure time for 8000 frames using a fluorescence microscope setup.^55^

#### DNA-PAINT with nanobodies

THP-1 caspase-1 knock-out cells imaged by DNA-PAINT were first labeled with a low amount of Alexa Fluor 647-conjugated anti-ASC nanobody to facilitate identification of the speck. Subsequently, the nanobody conjugated to a P3 docking strand was applied and the structure was imaged using the complementary imager strand labeled with Cy3B at 0.5 nM concentration in the above-mentioned imaging system. Images were recorded for 20,000–30,000 frames with an exposure time of 100 ms and a laser power density of ∼200 kW/cm^2^.

#### Dual-color DNA-PAINT imaging of THP-1 caspase-1 knock-out cells against ASC and beta-Tubulin

THP-1 caspase-1 knock-out cells were treated for NLRP3 inflammasome activation and fixed as described above. Cells were quenched by applying 200 mM NH4Cl (254134, Sigma-Aldrich) in PBS for 5 min, washed twice with PBS and permeabilized with 0.1% Triton X-100 (93443, Sigma-Aldrich) in PBS for 5 min. Subsequently, the sample was blocked for 1 h at room temperature using a blocking buffer containing 1xPBS, 3% BSA (9048468, Sigma-Aldrich), 1 mM EDTA (AM9260G), 0.02% Tween 20 (P1379, Sigma-Aldrich), and 0.05 mg/mL salmon sperm DNA (15632011). All antibodies were diluted in the above-mentioned blocking buffer. The sample was incubated overnight with purified monoclonal mouse anti-human ASC antibodies (TMS-1) (clone HASC-71) (Biolegend) at a final concentration of 33.5 nM (5 μg/mL) and primary rabbit β-tubulin antibody (5346, Cell Signaling Technologies, Danvers, Massachusetts, USA). As secondary probe, an anti-mouse nanobody conjugated to a P3-ATTO 488 docking strand (TTCTTCATTA-ATTO 488) (gift from Massive Photonics GmbH, Gräfelfing, Germany) was applied for 1 h at room temperature, which enabled diffraction-limited detection of the speck. To enable diffraction-limited detection of microtubules followed by DNA-PAINT imaging on selected cells, a secondary anti-rabbit Alexa Fluor 647-labeled nanobody was simultaneously applied with an anti-rabbit polyclonal antibody conjugated to a 3xR3 docking strand (CTCTCTCTCTC) (gift from Massive Photonics GmbH) at a final concentration of 10 nM and 33.3 nM (5 μg/mL), respectively and incubated for 1 h at room temperature. For fiducials, gold nanoparticles (G-90-100, cytodiagnostics, Burlington, Canada) were diluted 1:3 in PBS and incubated for 5 min at room temperature followed by a washing step with PBS. Samples were imaged by HiLo illumination using the DNA-PAINT microscope described above (either equipped with a Zyla sCMOS camera (Andor – Oxford Instruments, Belfast, Ireland) or a Hamamatsu Fusion BT camera (Hamamatsu, Japan) using an imager strand concentration between 100 and 500 p.m. in imaging buffer (1× PBS, 1 mM EDTA (AM9260G) and 500 mM NaCl (AM9759), pH 7.4); supplemented with 1× trolox (238813, Sigma-Aldrich) (P3-Cy3B imager strand: AATGAAGA-Cy3B; R3-Cy3B imager strand: GAGAGAG-Cy3B (Eurofins, Luxemburg)) and a 561 nm laser at a power of between 20 and 30 mW measured at the backfocal plane of the objective. Approximately 40,000 frames at 150 ms exposure time were recorded per channel. Gold beads were removed manually from the reconstructed images.

Super-resolution DNA-PAINT images were reconstructed using the Picasso^55^ and rendered with a Gaussian blur of one pixel corresponding to 13 nm.

### Quantification and statistical analysis

The details about the quantifications made and the statistical analyses are given in the figure legends or described in the method details above.
