# Supplementary material for: Nanoscale organization of the endogenous ASC speck
Source: iScience. 2023 Nov 2;26(12):108382. doi: 10.1016/j.isci.2023.108382 (PMC10690566; doi:10.1016/j.isci.2023.108382)
Supplement: Document S1. Figures S1–S14 [file mmc1.pdf]

iScience, Volume *26*

Supplemental information

Nanoscale organization of the endogenous ASC speck

Ivo M. Glück, Grusha Primal Mathias, Sebastian Strauss, Virgile Rat, Irene Gialdini, Thomas Sebastian Ebert, Che Stafford, Ganesh Agam, Suliana Manley, Veit Hornung, Ralf Jungmann, Christian Sieben, and Don C. Lamb

Table of Contents

Supplementary Figure S1: Diffraction-limited images of fluorescently labeled ASC in unstimulated and stimulated THP-1 cells and of extracellular ASC specks.. **S3**

Supplementary Figure S2: Applied image data analysis workflow.. **S4**

Supplementary Figure S3: The DBSCAN analysis **S5**

Supplementary Figure S4: Two-dimensional super-resolution images of endogenous ASC specks. **S6**

Supplementary Figure S5: Three-dimensional super-resolution images of endogenous ASC specks.. **S7**

Supplementary Figure S6: Dual-color super resolution images of Nigericin-treated THP-1 cells stained against ASC and beta-tubulin... **S8**

Supplementary Figure S7: Two-dimensional renderings of 3D super-resolution images of endogenous ASC specks **S9**

Supplementary Figure S8: Nanobody labeling of endogenous ASC specks **S10**

Supplementary Figure S9: Two-dimensional renderings of nanobody-labeled endogenous ASC specks imaged in three-dimensions using dSTROM.. **S11**

Supplementary Figure S10: Two-color super-resolution images of endogenous ASC specks **S12**

Supplementary Figure S11: Correlation plots of various parameters obtained from analysis of single cells .... **S13 - S14**

Supplementary Figure S12: Scatter plots corrections **S15**

Supplementary Figure S13: Controls for the applied antibody and F(ab')$_2$ fragment staining in THP-1 knock-out (KO) cells **S16**

Supplementary Figure S14: Controls for the applied nanobody (AF647-conjugated) staining in THP-1 knock-out (KO) cells **S17**

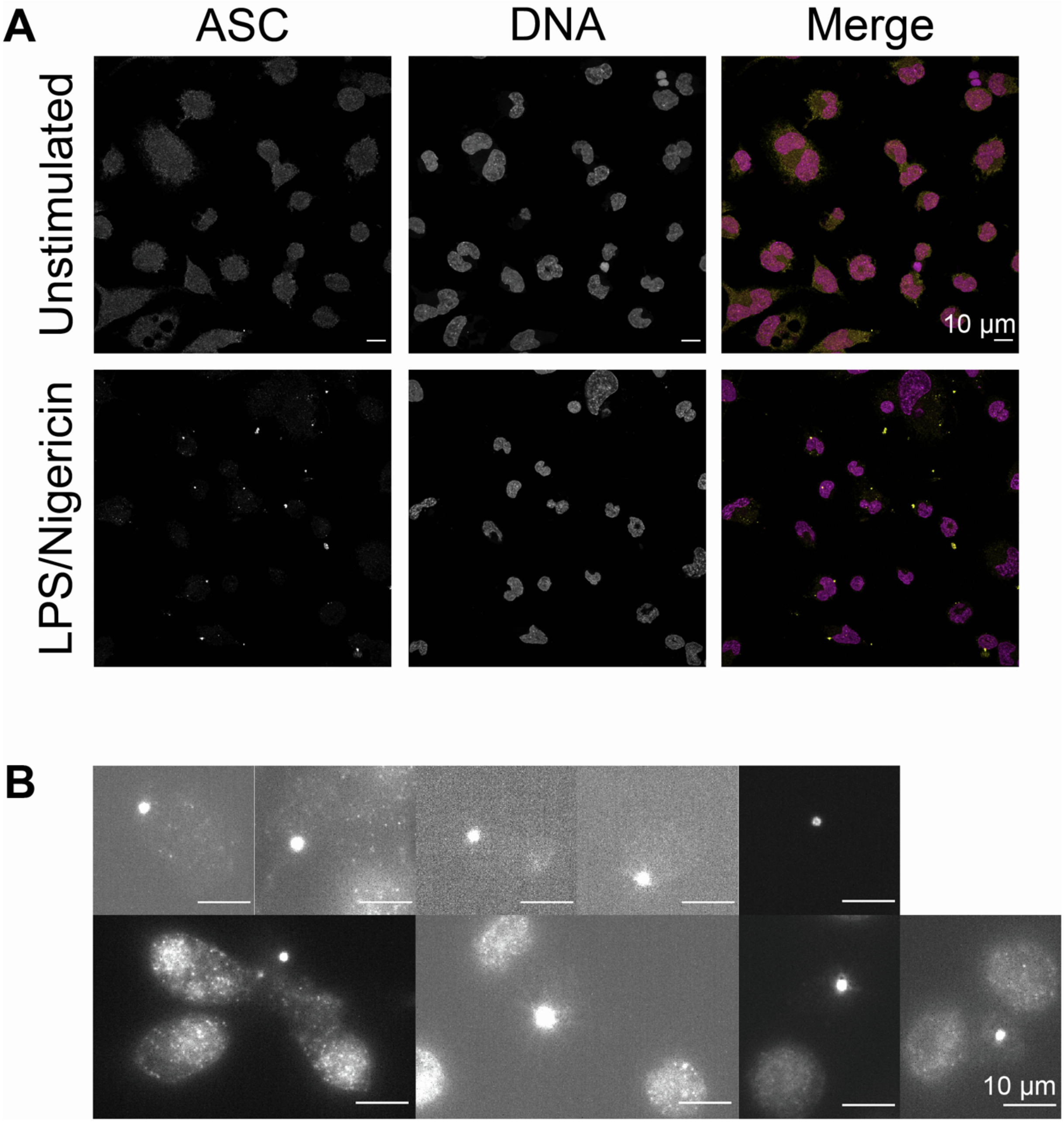

Supplementary Figure S1, related to Figure 1: Diffraction-limited images of fluorescently labeled ASC in unstimulated and stimulated THP-1 cells and of extracellular ASC specks. A) Confocal images (maximum projections) of ASC stained with primary mouse anti-human ASC antibody and secondary goat anti-mouse Alexa Fluor 647-conjugated F(ab')$_2$ fragments in unstimulated (upper panel) and in LPS + Nigericin-stimulated THP-1 cells (lower panel). Images are similar to those shown in Figure 1. B) Widefield images of extracellular ASC specks stained with primary mouse anti-human ASC antibody and secondary goat anti-mouse Alexa Fluor 647-conjugated F(ab')$_2$ fragments.

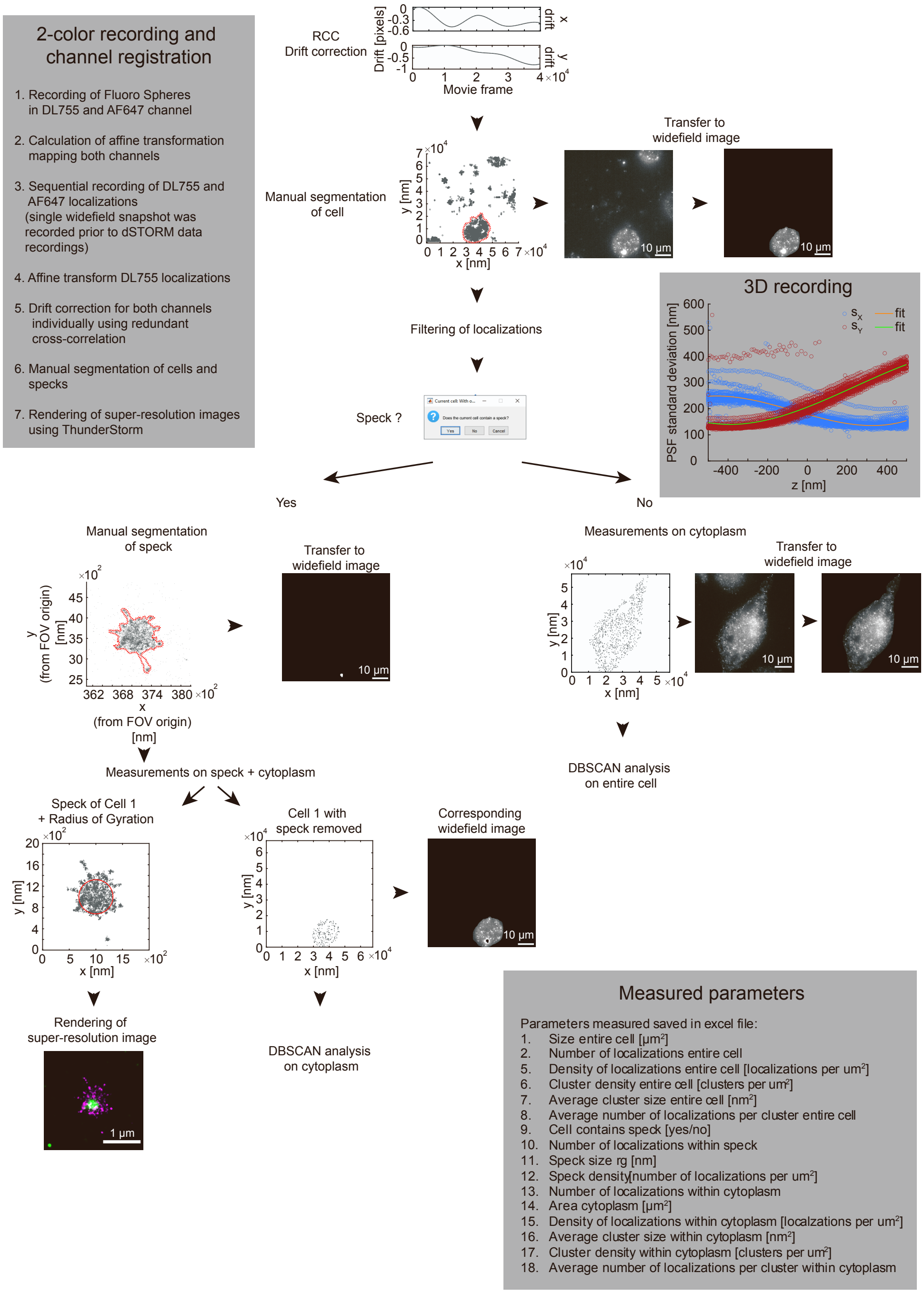

Supplementary Figure S2, related to Figures 2, 5 and 7: Applied image data analysis workflow. Widefield and dSTORM Microscopy experiments were performed on fixed cells. After localizing individual molecules and performing drift correction, the cells were manually segmented from the surroundings using the localizations and the obtained mask was applied to the widefield image. The localizations were filtered to remove unspecific and low-quality localizations. In cells containing an ASC speck, the speck was manually segmented and the obtained mask was applied to the widefield image. A super-resolution image was rendered from the localizations. Finally, various parameters for the entire cell, the cytosol and the ASC speck were calculated (listed in the box in lower, right corner). Cytosolic localizations were analyzed using the DBSCAN analysis (Supplementary Figure S3). The axial positions for 3D images were obtained by recording and fitting a calibration curve measured on TetraSpeck Microspheres (see gray box to the upper right). The 2-channel registration was performed as described in the box in the upper left. RCC: Redundancy cross-correlation.

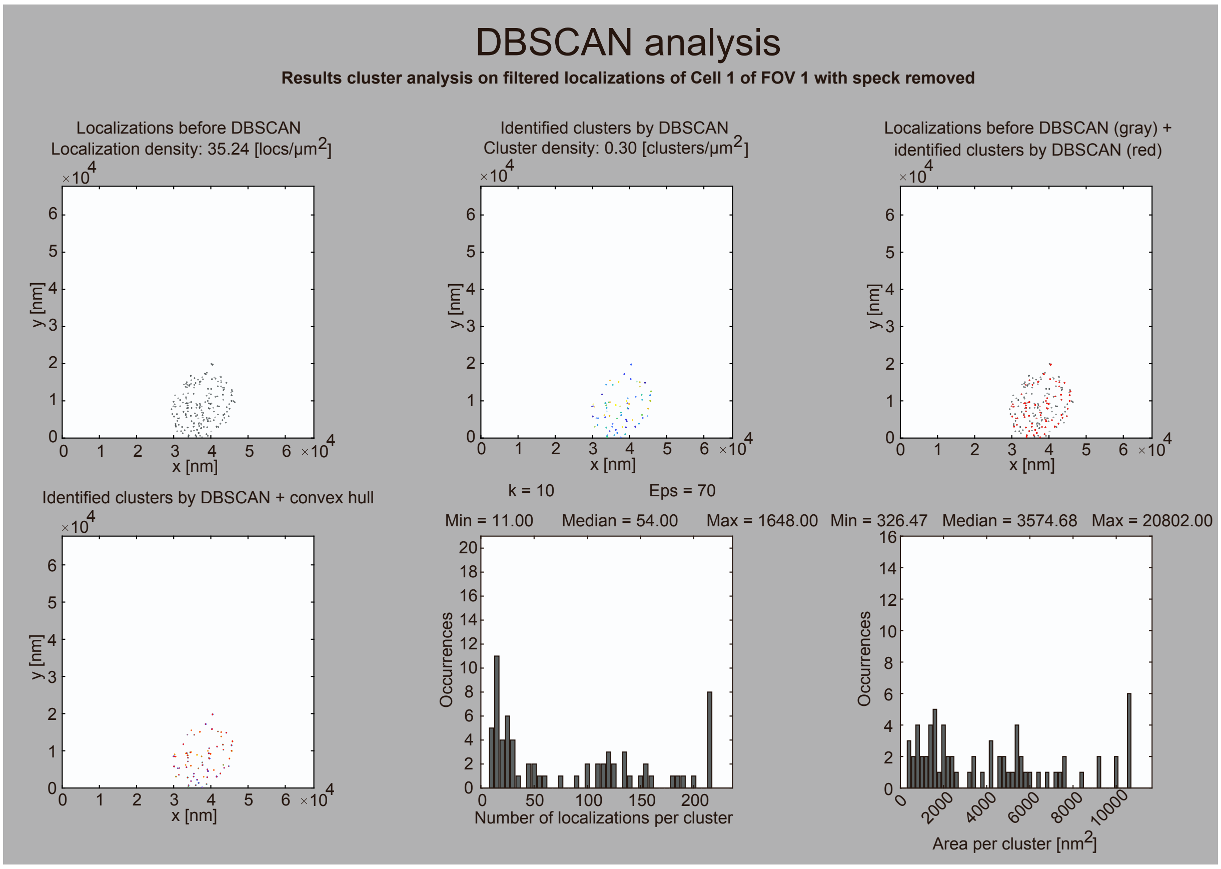

Supplementary Figure S3, related to Figure 2: The DBSCAN analysis. A screen shot of the DBSCAN analysis of the cytosolic non-speck bound ASC signal of the cell shown in Supplementary Figure S2 is depicted. The localization density before DBSCAN is shown in the top left panel. The top middle panel depicts the identified clusters after an analysis using a search radius of 70 nm and 10-300 localizations per cluster. Individual clusters are color-coded. The top right panel depicts an overlay of the identified clusters (red) with the unclustered localizations (gray). The bottom left panel depicts the clusters together with their convex hull. The bottom middle and right panels show histograms of the number of localizations per cluster and the area per cluster derived by calculation of the convex hull, respectively.

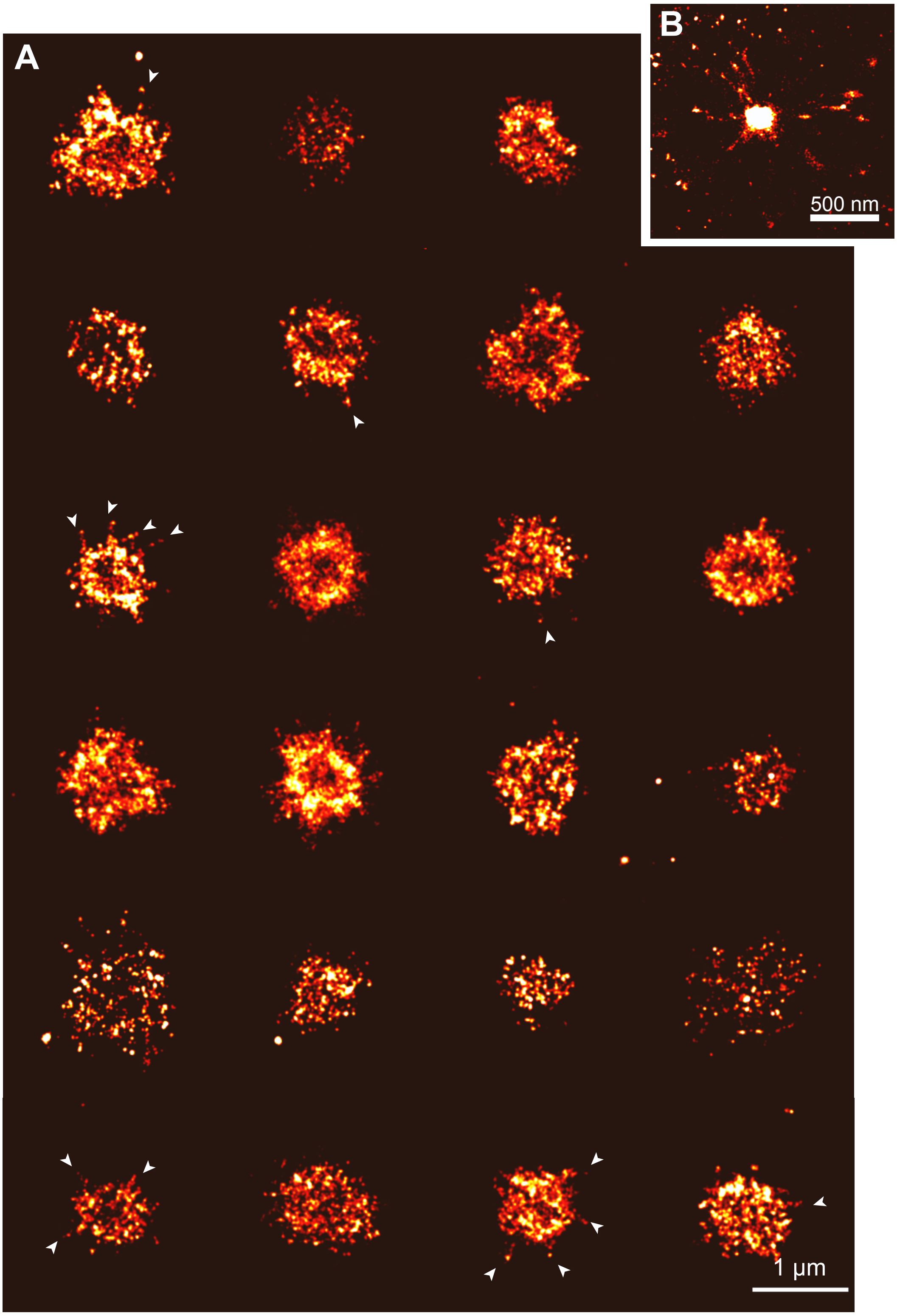

Supplementary Figure S4, related to Figures 3 and 5: Two-dimensional super-resolution images of endogenous ASC specks. A) A gallery of endogenous ASC specks labeled with a primary mouse anti-human ASC antibody and a secondary goat anti-mouse Alexa Fluor 647-conjugated F(ab')$_2$ fragment in THP-1 cells imaged using dSTORM. Some structures appear as ring-like structures and other as dense amorphous complexes. In several structures, filamentous extensions are observed, which are indicated by the arrows. B) An ASC speck in a BlaER1 cell observed using DNA-PAINT labeled with an ASC antibody and a secondary antibody conjugated with a P3 docking strand for DNA-PAINT imaging. Data was obtained on three independent cell preparations.

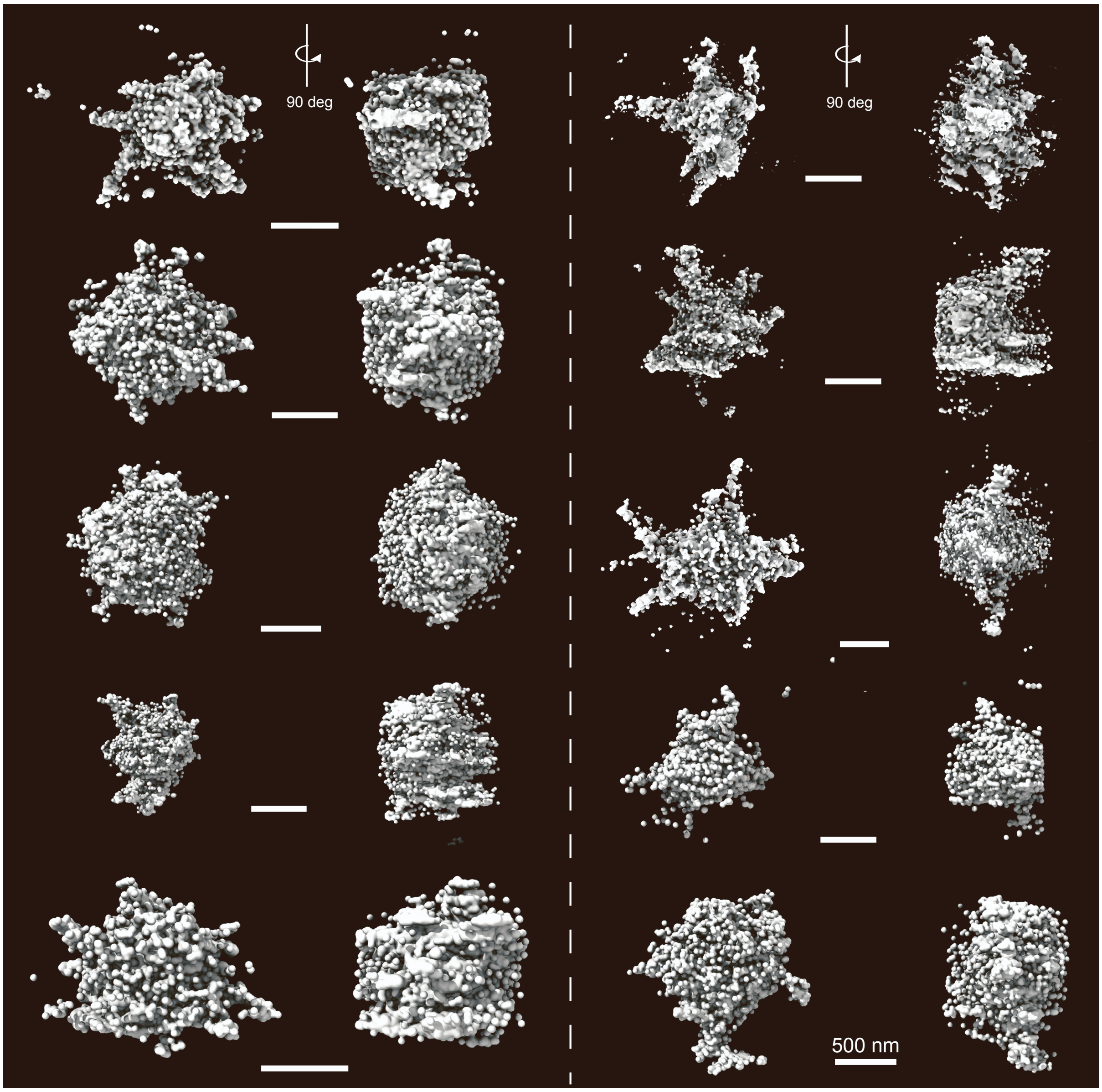

Supplementary Figure S5, related to Figure 3: Three-dimensional super-resolution images of endogenous ASC specks. 3D renderings of ASC specks that were stained using a primary mouse anti-human ASC antibody and a secondary goat anti-mouse Alexa Fluor 647-conjugated F(ab')$_2$ fragment and measured using dSTORM. Data were obtained on two independent cell preparations.

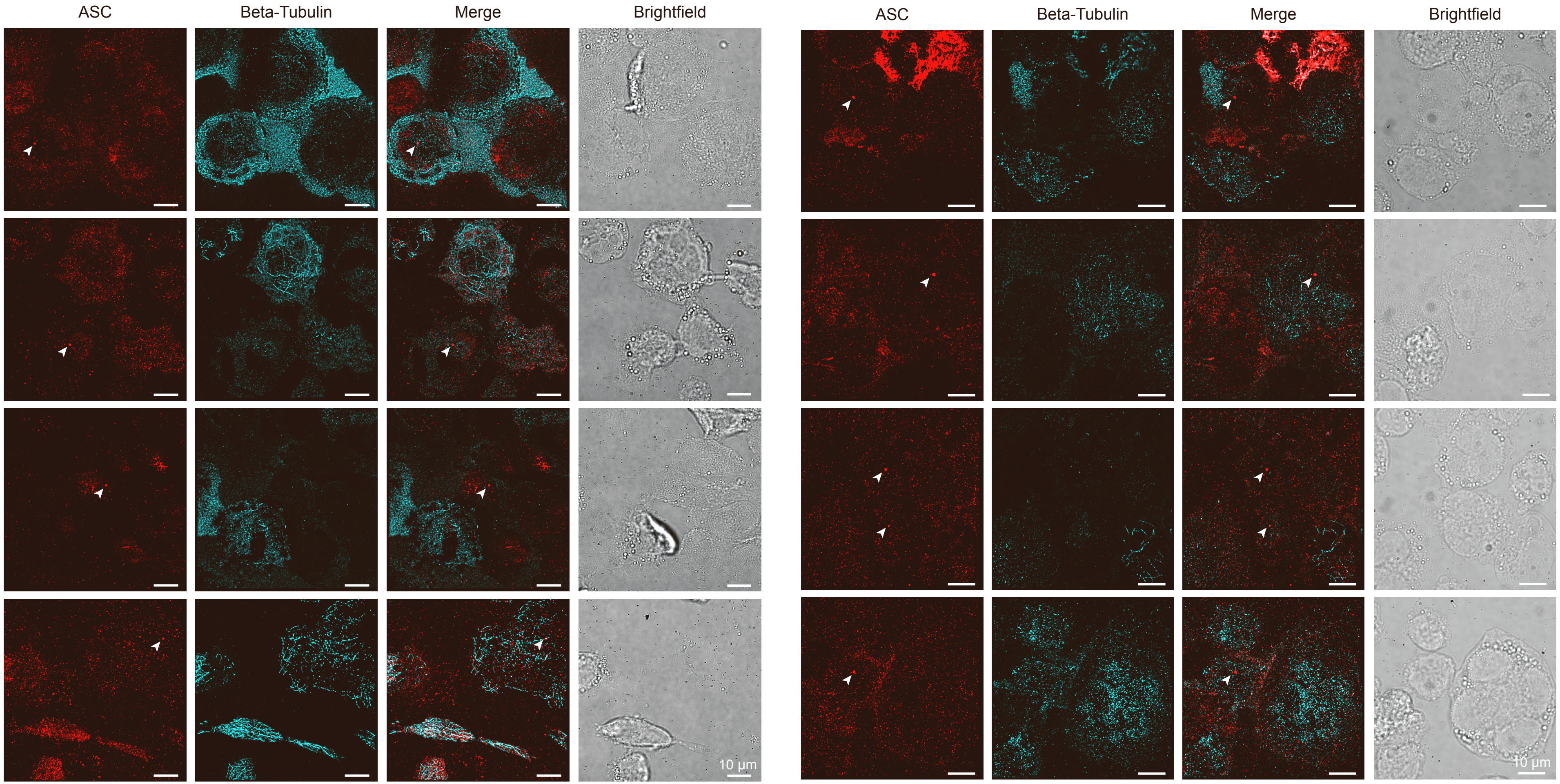

Supplementary Figure S6, related to Figure 3: Dual-color super-resolution images of Nigericin-treated THP-1 cells stained against ASC and β-tubulin. Dual-color DNA-PAINT images of THP-1 cells stained against ASC using a primary mouse anti-human ASC and secondary P3-labeled anti-mouse antibody (left images). ASC specks are highlighted by the white arrows. β-tubulin was stained using a primary rabbit anti-β-tubulin antibody and a secondary anti-rabbit R3-labeled antibody (2nd column). Merged images are shown in the third row followed by DIC images. The vast majority of microtubule filaments have disassembled in the cells.

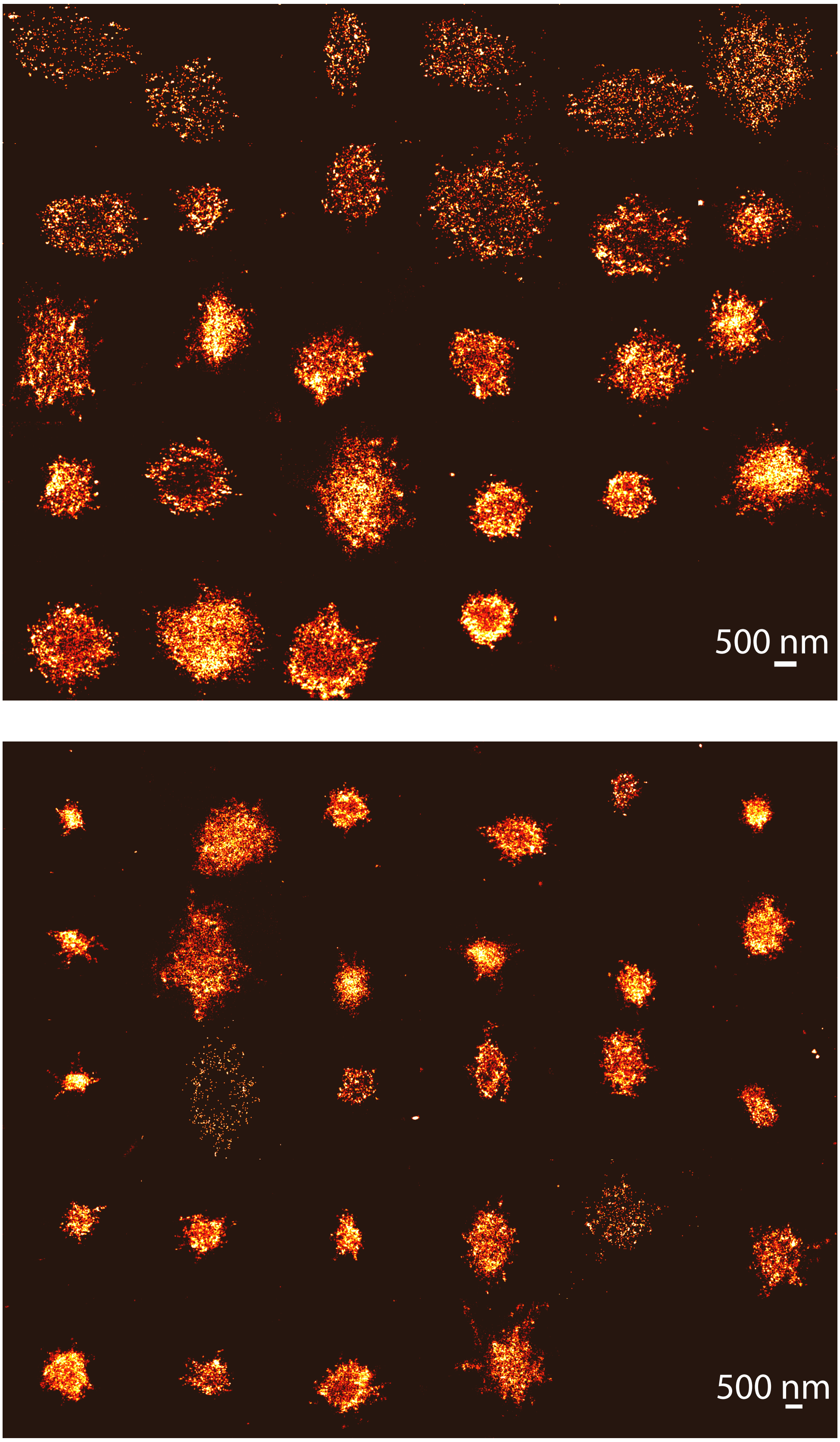

Supplementary Figure S7, related to Figures 3 and 5: Two-dimensional renderings of 3D super-resolution images of endogenous ASC specks. The ASC specks were measured in 3D using dSTORM and stained with a primary mouse anti-human ASC antibody and a secondary goat anti-mouse Alexa Fluor 647-conjugated secondary F(ab')$_2$ fragment. Data was obtained on two independent cell preparations.

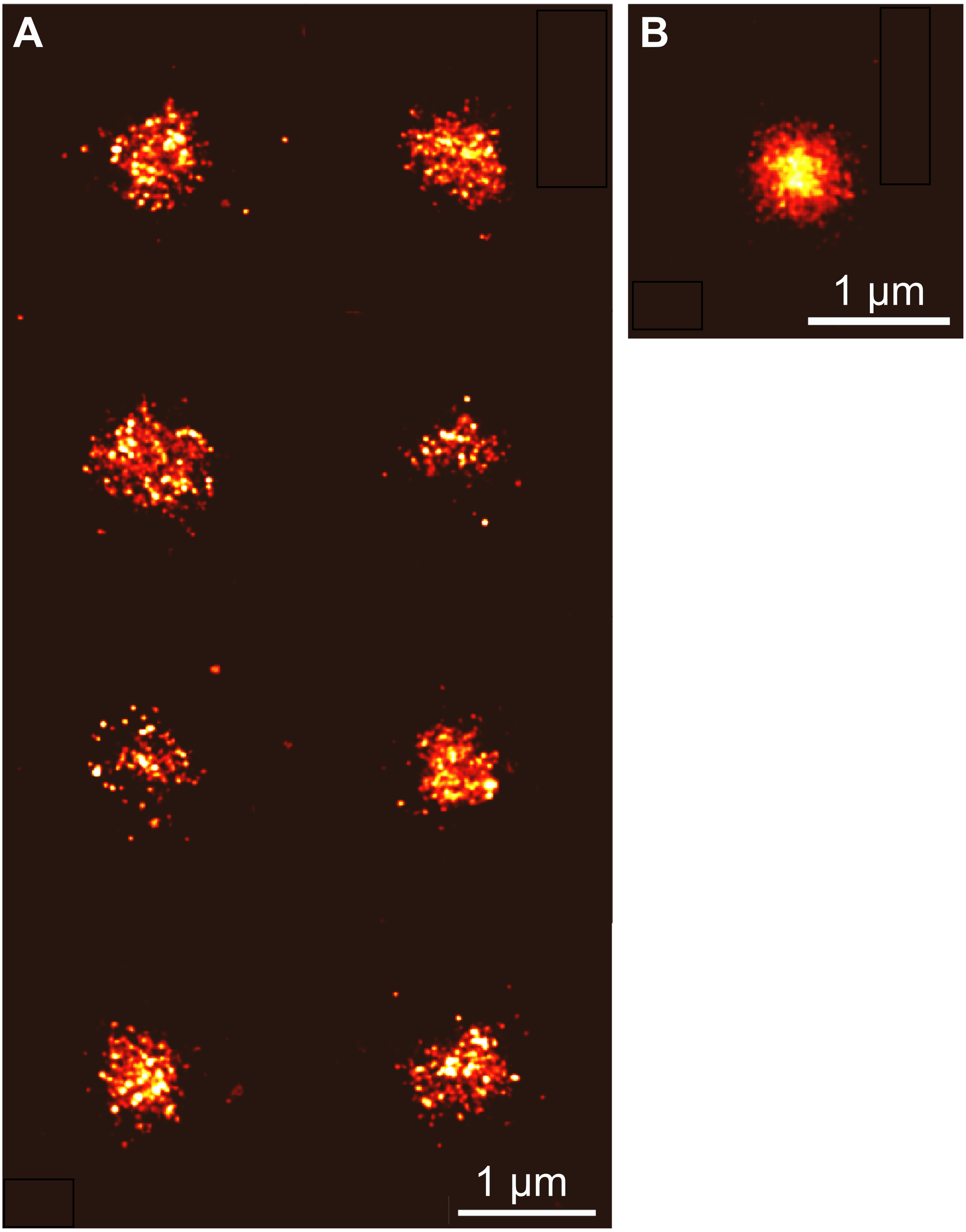

Supplementary Figure S8, related to Figure 5: Nanobody labeling of endogenous ASC specks. A) ASC specks in THP-1 cells were stained with Alexa Fluor 647-conjugated nanobody and imaged using 2D STORM. Data was obtained on a single cell preparation. B) An ASC speck in a THP-1 cell stained with a P3 DNA-PAINT docking strand-conjugated nanobody and imaged by 2D DNA-PAINT.

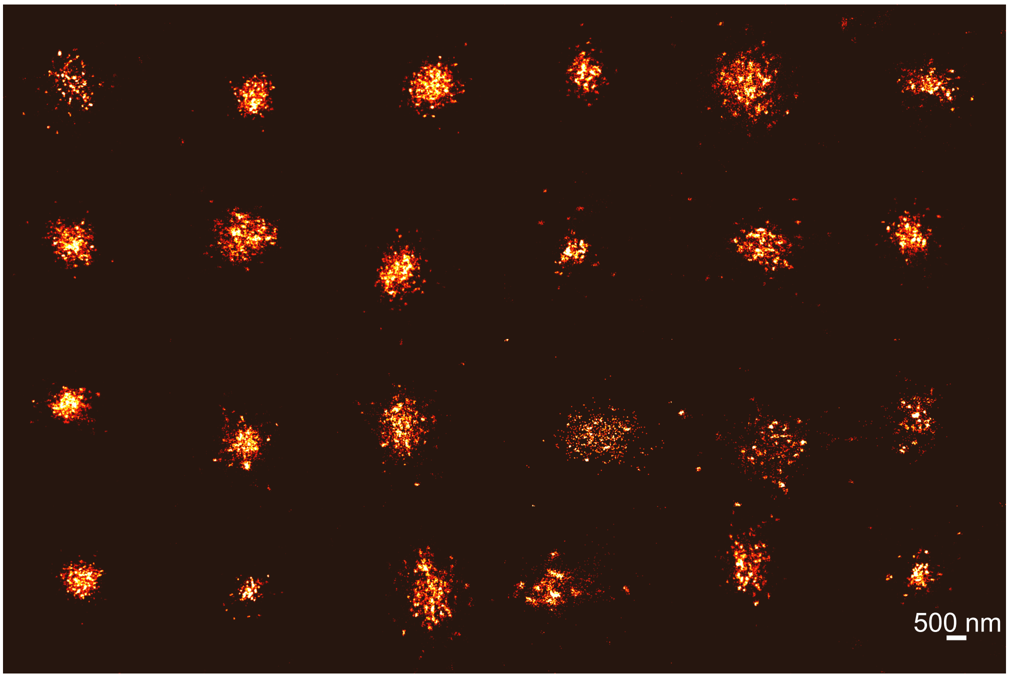

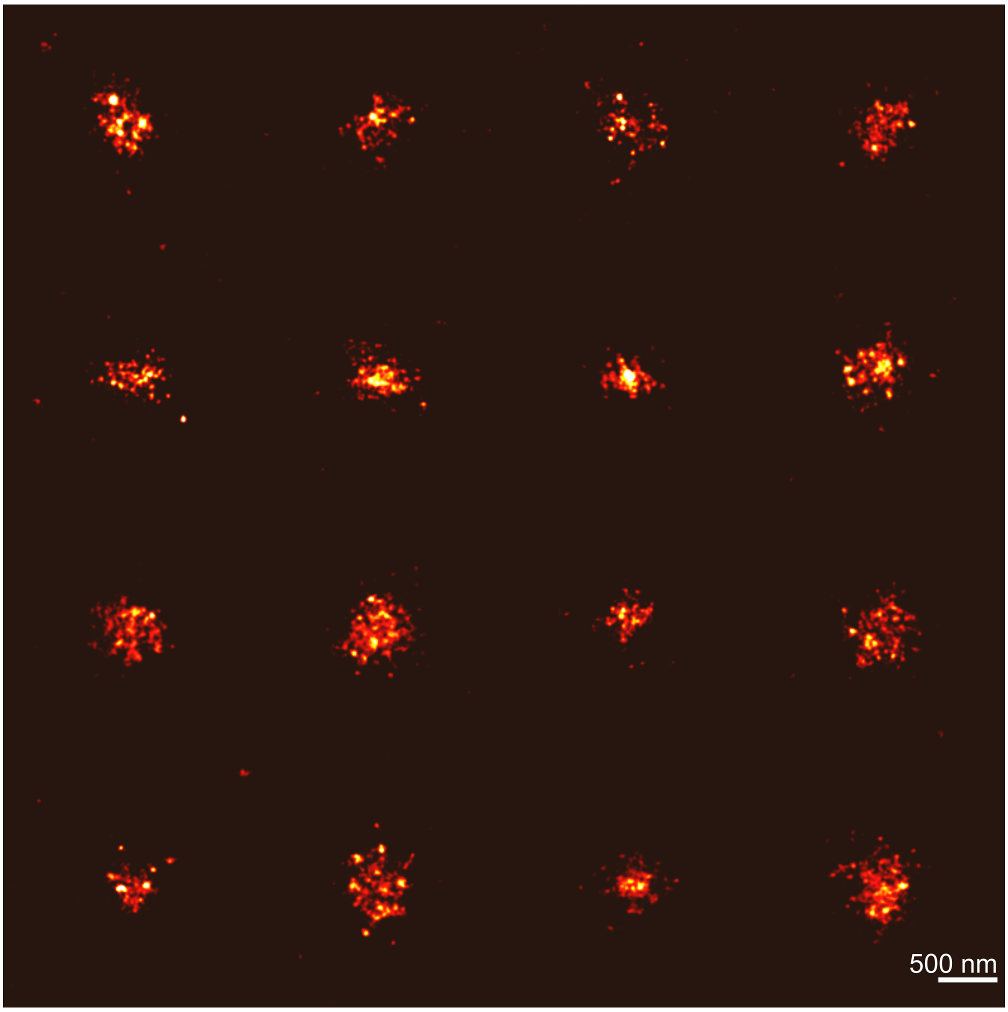

Supplementary Figure S9, related to Figure 5: Two-dimensional renderings of nanobody-labeled endogenous ASC specks imaged in three-dimensions using dSTROM. The ASC nanobody was conjugated with Alexa Fluor 647.

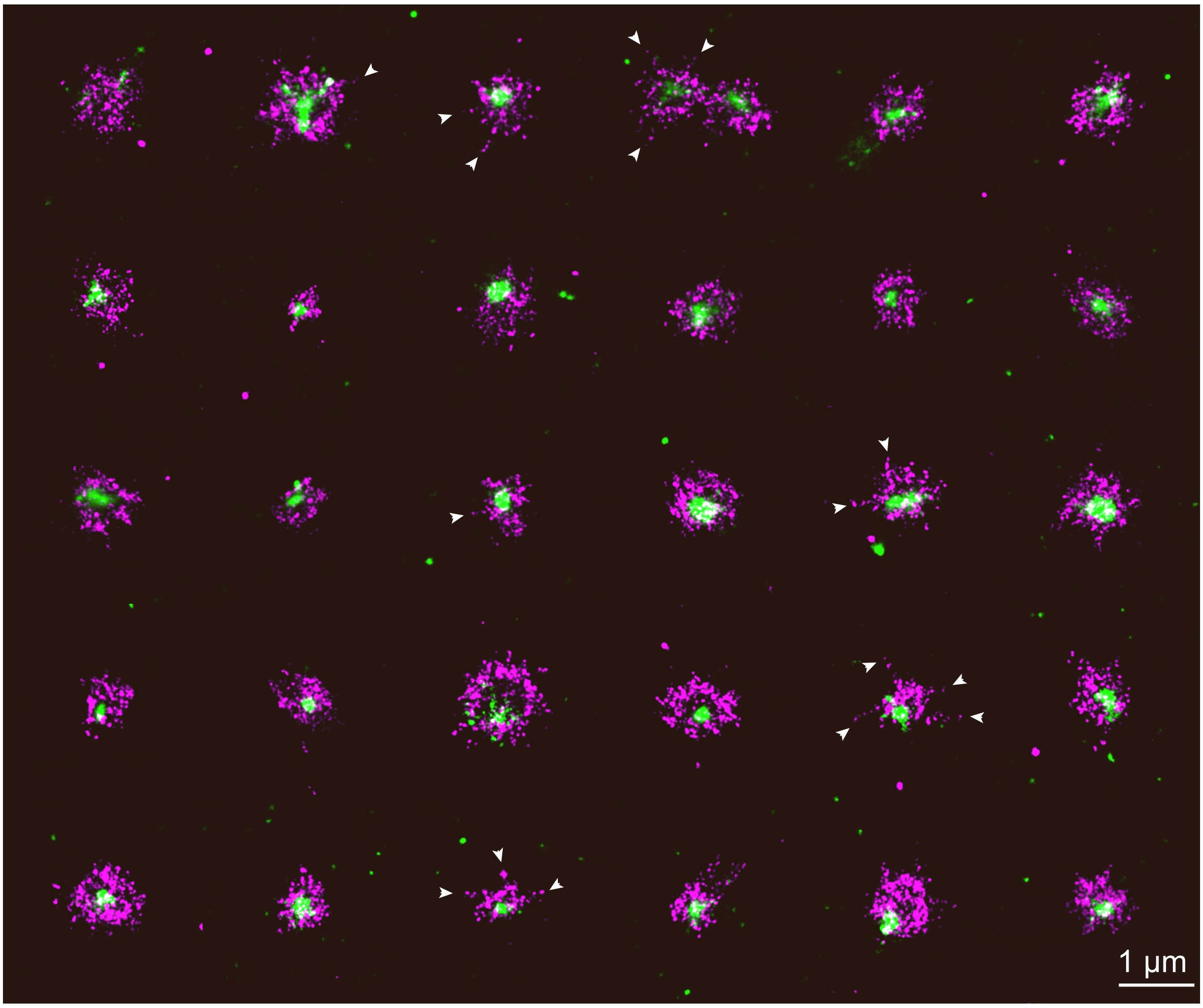

Supplementary Figure S10, related to Figure 6: Two-color, super-resolution images of endogenous ASC specks. 2D STORM images were collected of ASC specks labeled with primary mouse anti-human ASC antibody and secondary goat anti-mouse Alexa Fluor 647-conjugated F(ab')$_2$ fragment (magenta), and with DyLight 755-conjugated ASC nanobody (green). Arrows indicate filamentous extensions reaching out from the speck center. Data was obtained on a single cell preparation.

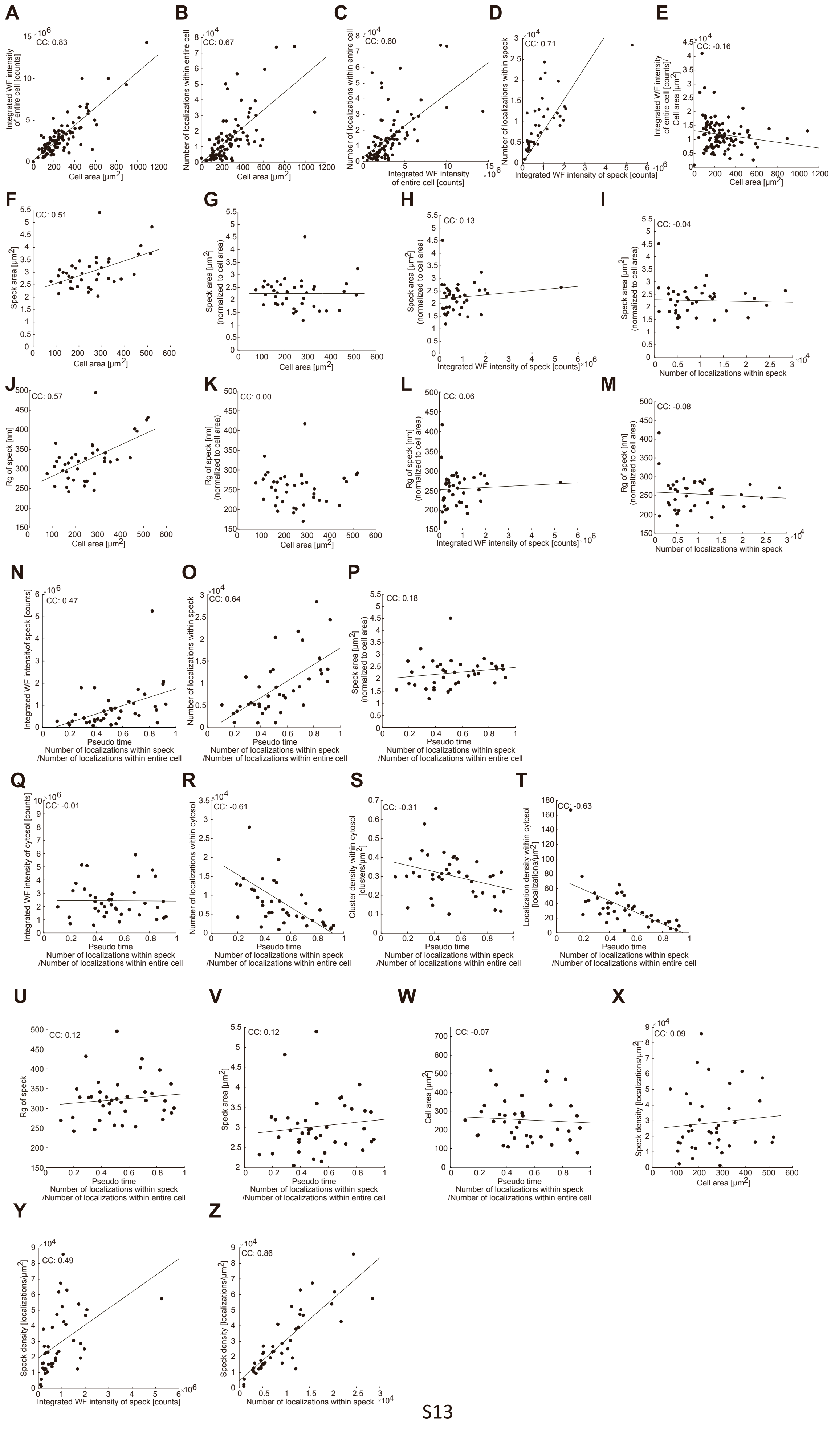

A
CC: 0.83
Integrated WF intensity of entire cell [counts]
Cell area [μm2]
B
CC: 0.67
Number of localizations within entire cell
Cell area [μm2]
C
CC: 0.60
Number of localizations within entire cell
Integrated WF intensity of entire cell [counts]
D
CC: 0.71
Number of localizations within speck
Integrated WF intensity of speck [counts]
E
CC: -0.16
Integrated WF intensity of entire cell [counts]/ Cell area [μm2]
Cell area [μm2]
F
CC: 0.51
Speck area [μm2]
Cell area [μm2]
G
Speck area [μm2] (normalized to cell area)
Cell area [μm2]
H
CC: 0.13
Speck area [μm2] (normalized to cell area)
Integrated WF intensity of speck [counts]
I
CC: -0.04
Speck area [μm2] (normalized to cell area)
Number of localizations within speck
J
CC: 0.57
Rg of speck [nm]
Cell area [μm2]
K
CC: 0.00
Rg of speck [nm] (normalized to cell area)
Cell area [μm2]
L
CC: 0.06
Rg of speck [nm] (normalized to cell area)
Integrated WF intensity of speck [counts]
M
CC: -0.08
Rg of speck [nm] (normalized to cell area)
Number of localizations within speck
N
CC: 0.47
Integrated WF intensity of speck [counts]
Pseudo time
Number of localizations within speck /Number of localizations within entire cell
O
CC: 0.64
Number of localizations within speck
Pseudo time
Number of localizations within speck /Number of localizations within entire cell
P
CC: 0.18
Speck area [μm2] (normalized to cell area)
Pseudo time
Number of localizations within speck /Number of localizations within entire cell
Q
CC: -0.01
Integrated WF intensity of cytosol [counts]
Pseudo time
Number of localizations within speck /Number of localizations within entire cell
R
CC: -0.61
Number of localizations within cytosol
Pseudo time
Number of localizations within speck /Number of localizations within entire cell
S
CC: -0.31
Cluster density within cytosol [clusters/μm2]
Pseudo time
Number of localizations within speck /Number of localizations within entire cell
T
CC: -0.63
Localization density within cytosol [localizations/μm2]
Pseudo time
Number of localizations within speck /Number of localizations within entire cell
U
CC: 0.12
Rg of speck
Pseudo time
Number of localizations within speck /Number of localizations within entire cell
V
CC: 0.12
Speck area [μm2]
Pseudo time
Number of localizations within speck /Number of localizations within entire cell
W
CC: -0.07
Cell area [μm2]
Pseudo time
Number of localizations within speck /Number of localizations within entire cell
X
CC: 0.09
Speck density [localizations/μm2]
Cell area [μm2]
Y
CC: 0.49
Speck density [localizations/μm2]
Integrated WF intensity of speck [counts]
Z
CC: 0.86
Speck density [localizations/μm2]
Number of localizations within speck

Supplementary Figure S11, related to Figure 7: Correlation plots for various parameters obtained from the analysis of single cells. Panels A – E relate the number of localizations, the widefield intensity and the cell area with each other. Panels F – I depicts how the size of the speck depends on cell area and ASC concentration in the speck. Panels J - M depicts how the radius of gyration Rg, depends on cell area and ASC concentration in the speck. Panels N – X depict how various parameters depend on the pseudo time. Specifically, the amount of ASC in the speck (N, O), the speck area (P), the amount of ASC in the cytosol (Q, R), the cytosolic cluster density (S) and localization density (T), the radius of gyration, Rg (U), the speck area (V), the cell area (W) and the speck density (X). Panels Y – Z depict how the speck density depends on the amount of ASC in the speck. Data was obtained on three independent cell preparations. WF: widefield; CC: Pearson correlation coefficient

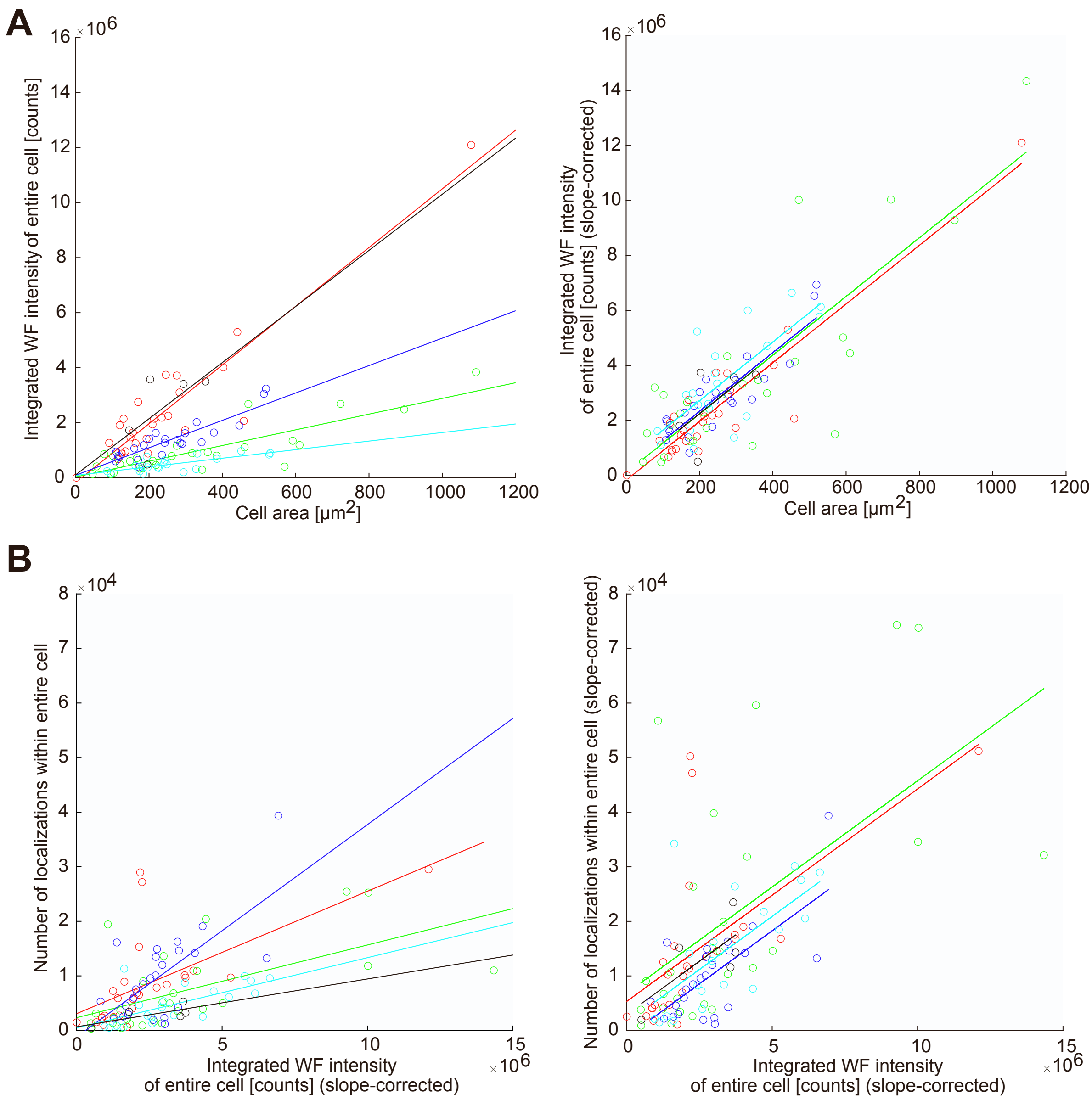

Supplementary Figure S12, related to Figure 7: Scatter plots corrections. The uncorrected and corrected scatter plots are shown to illustrate the correction to the integrated widefield intensity and localization data for differences between measurement days. Different sample preparations and dSTORM buffer conditions lead to variations in the widefield intensity and number of localizations with respect to ASC content. Hence, we used the correlation between ASC content and cell area to normalize the widefield intensity for different measurement days. For the number of localizations, we used the linear correlation between intensity and number of detected localizations. A) Scatter plot of the integrated widefield intensity of the entire cell against the cell area including the linear fits of individual measurement days (left panel) and the normalized integrated widefield intensity by adjusting the individual slopes to that of the measurement day with the steepest slope against the cell area (right panel). B) Scatter plot of the number of localizations within the entire cell against the normalized integrated widefield intensity of the entire cell including the linear fits of individual measurement days (left panel) and number of localizations within the entire cell normalized with respect to the measurement day with the steepest slope plotted against the normalized integrated widefield intensity of the entire cell (right panel) The individual measurement days are color-coded.

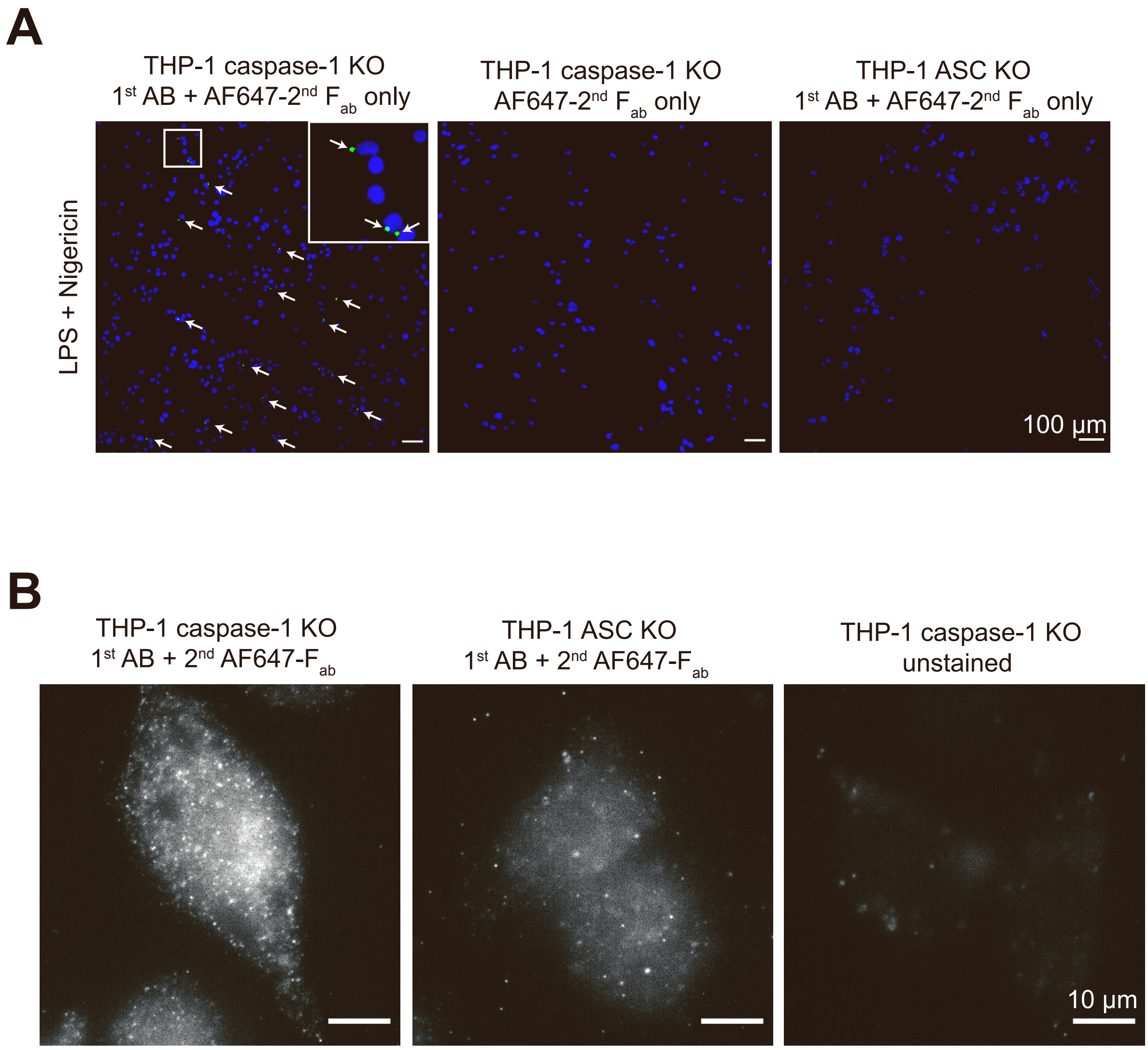

Supplementary Figure S13, related to Figures 1 - 8: Controls for the applied antibody and F(ab')$_2$ fragment staining in THP-1 knock-out (KO) cells. A) 10x overview images recorded on a confocal spinning disk microscope indicating the specificity of the applied ASC staining using a primary mouse anti-human ASC antibody and a secondary goat anti-mouse Alexa Fluor 647-conjugated F(ab')$_2$ fragment; green: Alexa Fluor 647 (summed projections of a recorded Z-stack are shown); blue: DNA-staining with DAPI. No ASC staining was observed in the absence of ASC or primary antibody. B) Higher magnification images (60x) verifying the specificity of ASC staining.

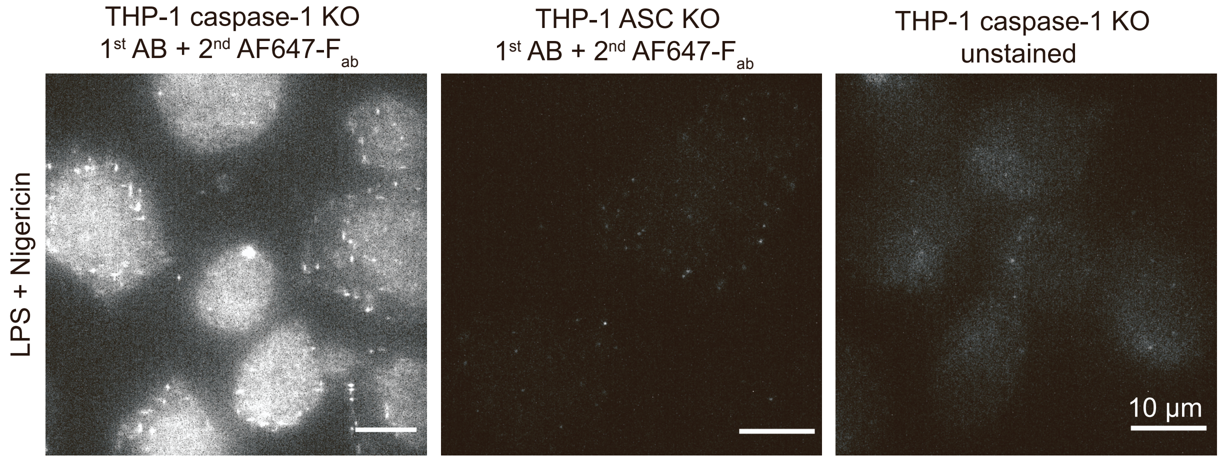

Supplementary Figure S14, related to Figures 5, 6, 7 and 8: Controls for the applied nanobody (AF647-conjugated) staining in THP-1 knock-out (KO) cells. Widefield images recorded at 60x magnification are shown. Stimulated and labeled cells are shown in the left panel. No unspecific signal was observed in the absence of ASC (middle panel) or in unstained cells (right panel) verifying the specificity of ASC staining. All images are displayed using the same intensity scale.
